# Accurate prediction of sepsis from pediatric emergency department to PICU using a machine-learning model

**DOI:** 10.3389/fped.2025.1610187

**Published:** 2025-10-10

**Authors:** Xuan Shi, Xuying Wang, Haomei Yang, Xiaowei Fan, Guangming Liu, Yongling Song, Qiuyan Peng, Qiang Wang, Xin Sun, Wencheng Ma, Peiqing Li

**Affiliations:** ^1^ Pediatric Emergency Department, Guangzhou Women and Children’s Medical Center, Guangzhou Medical University, Guangdong Provincial Clinical Research Center for Child Health, Guangzhou, China; ^2^ Ewell Technology Company, Hangzhou, China; ^3^ Medical Department of Guangzhou Women and Children’s Medical Center, Guangzhou Medical University, Guangdong Provincial Clinical Research Center for Child Health, Guangzhou, China

**Keywords:** pediatric sepsis, early warning, machine learning, XGBoost, recurrent neural network, SHAP, electronic health records

## Abstract

**Background:**

Timely identification of pediatric sepsis remains a critical challenge in emergency and intensive care settings due to the heterogeneous clinical presentations across age groups. Existing scoring systems often lack temporal resolution and interpretability. We aimed to develop a real-time, machine learning–based prediction framework integrating static and dynamic electronic health record (EHR) features to support early sepsis detection.

**Methods:**

This retrospective study included pediatric patients from Guangzhou Women and Children's Medical Center (GWCMC; *n* = 1,697) and an external validation cohort from the MIMIC-III database (*n* = 827). Irregular time-series data were imputed using a correlation-enhanced continuous time-window histogram with multivariate Gaussian processes (CTWH + MGP). We compared the predictive performance of XGBoost and gated recurrent unit (GRU)-based RNN models over a 12-h window prior to clinical diagnosis. Model outputs were validated internally and externally using AUROC, AUPRC, and Youden index, with SHAP-based interpretability applied to identify key clinical features.

**Results:**

The CTWH + MGP-XGBoost model achieved the highest AUROC at diagnosis time (T = 0 h; AUROC = 0.915), while the GRU-based model demonstrated superior temporal stability across early windows. Top contributing features included lactate, white blood cell count, pH, and vasopressor use. External validation confirmed generalizability (MIMIC-III AUROC = 0.905). Simulation of real-time alerts showed a median lead time of 6.2 h before clinical diagnosis, with *κ* = 0.82 agreement against physician-confirmed cases.

**Conclusions:**

Our results suggest that a dual-model ensemble combining interpolation-based preprocessing and interpretable machine learning enables robust early sepsis detection in pediatric populations. The system supports integration into EHR platforms for real-time clinical alerts and may inform prospective trials and quality improvement initiatives.

## 1 Background

Sepsis remains one of the leading causes of morbidity and mortality in children worldwide, accounting for an estimated 25%–40% of pediatric intensive care unit (PICU) admissions and nearly 8% of in-hospital mortality globally (1, 2). Despite advances in antimicrobial therapy and critical care monitoring, early diagnosis of pediatric sepsis remains a persistent challenge due to the heterogeneity of clinical presentations, age-related physiological variability, and the non-specific nature of early warning signs (3–5). Current sepsis screening tools such as SIRS, qSOFA, and PELOD-2 often fail to achieve sufficient sensitivity or lead time in real-world pediatric emergency settings (6–8).

Artificial intelligence and machine learning techniques offer new opportunities to augment early sepsis detection by leveraging high-dimensional electronic health record (EHR) data in real time (9–11). Prior models have demonstrated promising results in adult cohorts using long short-term memory (LSTM), gradient boosting, and attention-based architectures (12, 13). However, limited work has translated these findings into pediatric populations, where data sparsity, missingness, and developmental variability pose unique modeling challenges (14, 15). Hemodynamic support in pediatric septic shock remains challenging, with current practice guided by the American College of Critical Care Medicine's parameters (3).

To address these issues, we developed a real-time prediction framework for pediatric sepsis integrating a correlation-enhanced continuous time-window histogram (CTWH) interpolation with multivariate Gaussian processes (MGP) (13, 14), combined with an ensemble of gradient boosting (XGBoost) and recurrent neural network (RNN) models (12, 16). The framework was trained and validated using two large pediatric datasets, including an internal cohort from a high-volume tertiary pediatric emergency department in China and an external validation cohort from the MIMIC-III database (17).

In this study, we aimed to assess the framework's predictive performance across various time windows preceding sepsis onset, evaluate feature interpretability using SHAP values, and simulate real-time deployment scenarios to explore clinical feasibility. We hypothesized that this interpolation-guided, dual-model ensemble would enhance early risk stratification and offer timely alerts suitable for integration into existing EHR systems.

## 2 Methods

**Figure 1:**
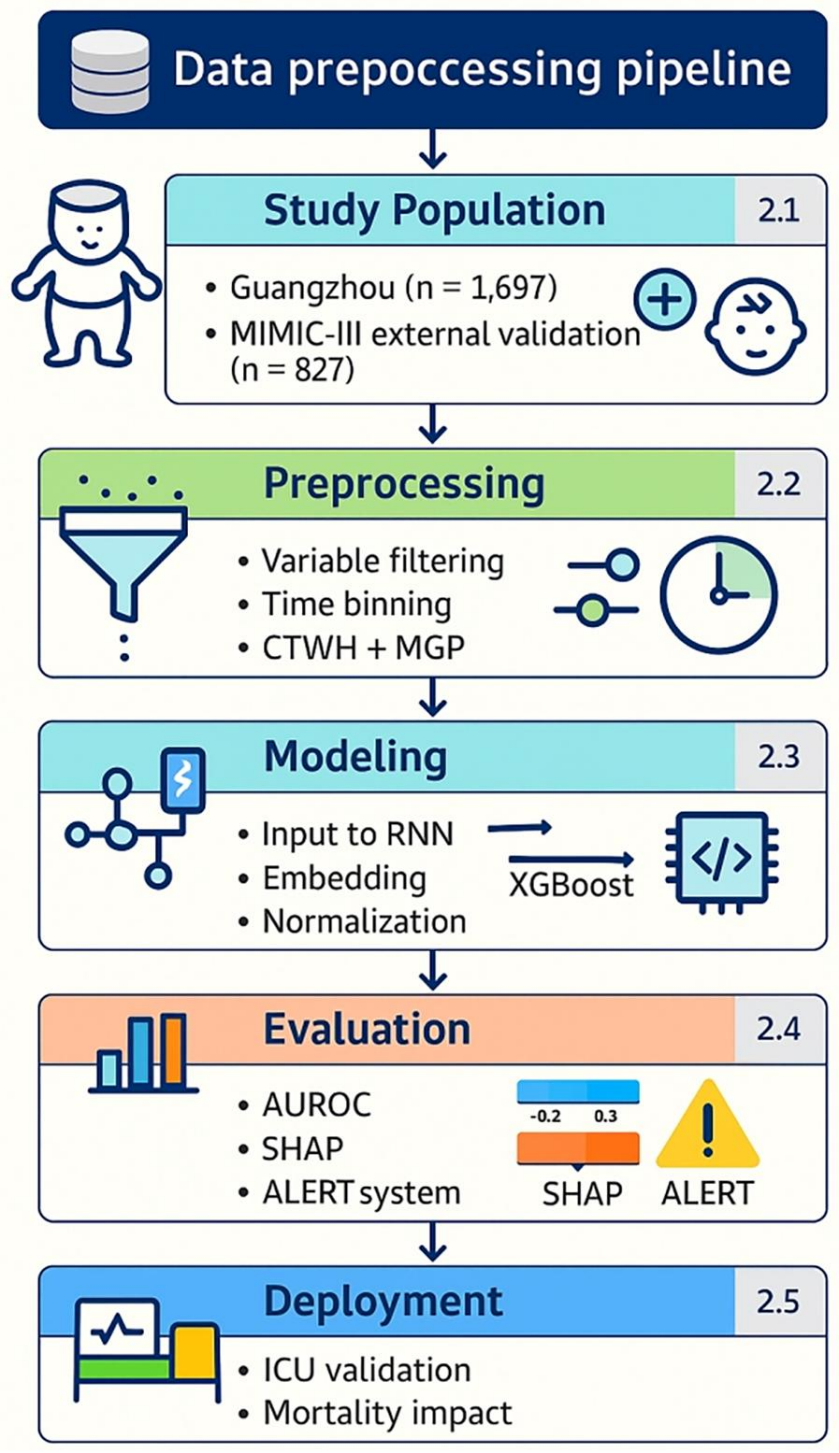
Overall workflow from dataset selection to model deployment. Outlines the full data preprocessing and modeling workflow, aligned with the structural segmentation of this study. Each module corresponds to a section in the Methods. Core indicators such as sample sizes, feature selection count, model performance (AUROC), and interpretability tools (SHAP) are embedded.

Figure 1 outlines the full data preprocessing and modeling workflow, aligned with the structural segmentation of this study (Sections 2.1–2.5). Overall workflow from dataset selection to model deployment. Each module corresponds to a section in the Methods. Core indicators such as sample sizes, feature selection count, model performance (AUROC), and interpretability tools (SHAP) are embedded.

### 2.1 Dataset description

This retrospective study included two pediatric cohorts. The internal cohort comprised 1,697 patients under 18 years of age admitted to Guangzhou Women and Children's Medical Center (GWCMC) from February 2016 to July 2018. Pediatric sepsis was identified based on modified Sepsis-III criteria (18), with adjustments for age and clinical presentation. The Sepsis-3 consensus definition provided a robust framework for identifying sepsis and septic shock, which we adapted for pediatric use in this study (19). Electronic health record (EHR) data were extracted, encompassing vital signs, laboratory results, medications, and nursing assessments within the 12-h window preceding a sepsis diagnosis. Cases with incomplete records, neonates, or acute upper respiratory infections were excluded.

**Figure 2:**
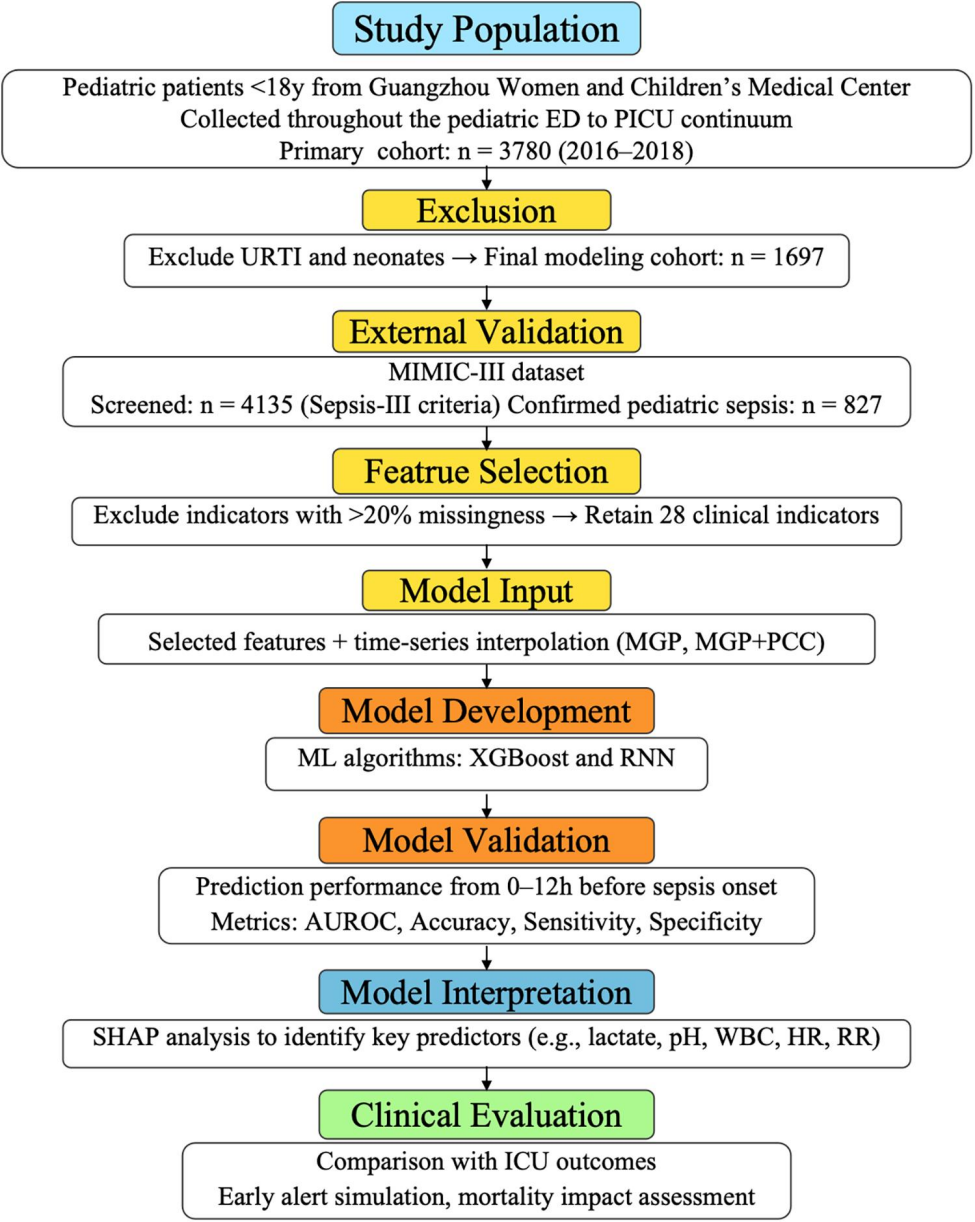
Inclusion and exclusion criteria for the Guangzhou women and Children's medical center dataset. This flowchart outlines the selection process for the internal pediatric cohort. A total of 3,780 children aged <18 years who visited the emergency department or were admitted to the PICU between February 2016 and July 2018 were initially screened. After excluding cases with neonatal status, acute upper respiratory tract infections, and incomplete electronic health record data, 1,697 patients with confirmed infection or sepsis-related diagnoses were included for model development and validation. The final cohort included both septic and non-septic patients, defined according to the pediatric adaptation of Sepsis-III criteria, with retrospective verification by senior pediatric intensivists.

The external validation cohort was derived from the MIMIC-III database, consisting of 827 pediatric ICU encounters fulfilling Sepsis-III criteria (17). Variable definitions and coding structures were harmonized across cohorts to ensure consistency. Figure 2 illustrate variable frequency and inter-variable correlations, respectively.

### 2.2 Data preprocessing

Variables with >20% missingness were excluded from model development. As summarized in Table 1 (Additional File 1), a total of 28 variables were retained, including core vital signs (HR, RR, SpO₂, T), key laboratory biomarkers (WBC, creatinine, lactate, pH), therapeutic indicators (antibiotics, glucocorticoids, vasopressors), and fluid balance measures. Sixteen variables (e.g., bilirubin, troponin, IL-6, PaCO₂) exceeded the 20% missingness threshold (25%–50%) and were therefore excluded. This structured selection ensured that retained features were both clinically relevant and statistically reliable.

**Table 1:** Size and number of interpolated windows (Guangzhou women and children's hospital dataset).

Index	T	HR	R	BSD	AC	GCS	SO2	MV	BG
Window size	5	4	4	22	20	19	14	20	12
Number of windows	1	1	1	2	1	17	1	1	1
Index	CRP	PO2	INR	WBC	PT	PLT	TB	LAC	Cr
Window size	3	5	24	26	24	28	22	14	32
Number of windows	1	2	1	4	1	2	2	2	2
Index	PH	AMON	ALP	K	CA	Mg	DBIL	FIB	HGB
Window size	10.3	15	21	15	15	27	22	24	3
Number of windows	2	1	1	1	3	1	2	1	3
Index	HCT	AST	SBP	DBP	MAP	HCO3	LAC	PCO2	FIB
Window size	4	27	9	10	10	20	20	22	75
Number of windows	3	2	3	1	1	2	1	3	2

This table presents the optimized interpolation window sizes (in hours) and corresponding number of windows across selected clinical variables in the Guangzhou Women and Children's Medical Center dataset. These parameters guided the correlation-assisted time-series imputation strategy. Window size is measured in hours. Variables with >20% missingness were excluded. The window number indicates how many historical segments were used for correlation referencing.

**Figure 3:**
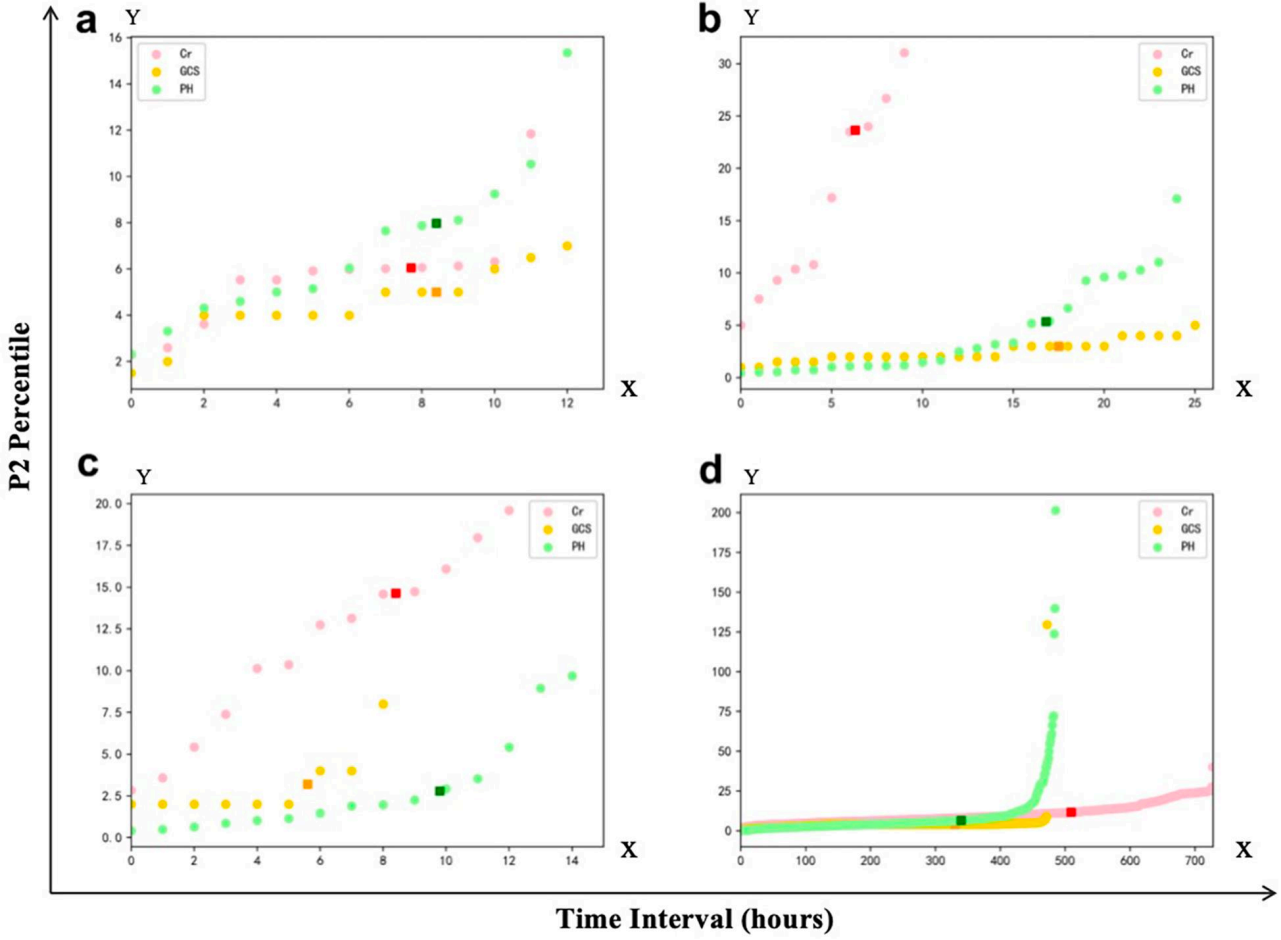
Representative examples of frequency distribution of different clinical variables over time in patients from the Guangzhou women and Children's medical center. **(a–c)** Represent the time interval distributions of three variables, i.e., creatinine (Cr), glucocorticoids (GCS), and Pondus Hydrogenii (PH), of three patients, respectively, where the vertical axis represents the time interval in hours. The darker point represents the P1 quantile (70% quantile) corresponding to this variable. **(d)** Represents the P1 quantile distribution of the three variables, corresponding to all the patients.

**Figure 4:**
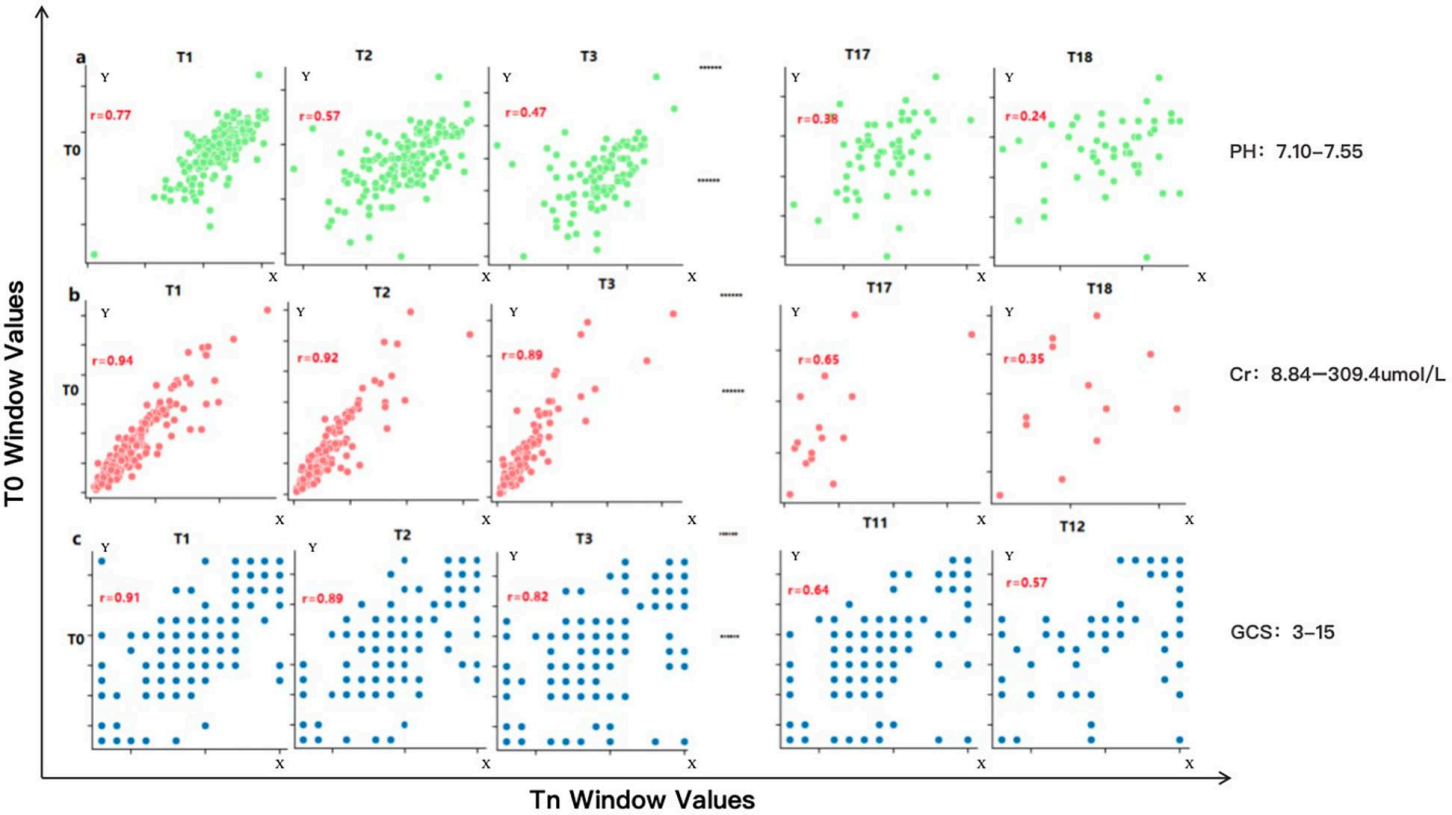
Correlation performance of different variables obtained from the Guangzhou women and Children's medical center dataset across different time windows. This figure illustrates the temporal autocorrelation for three representative clinical variables—serum creatinine (Cr), pH, and Glasgow Coma Scale (GCS)—using scatter plots between reference window T0 and successive windows (T1, T2, T3…). **(a)** pH shows weak correlation beyond one window (r < 0.6), allowing only short-range interpolation (∼20.6 h). **(b)** Cr retains moderate correlation across three windows, supporting interpolation up to 96 h. **(c)** GCS shows intermediate range stability with a correlation-informed interpolation span of 40 h. A correlation coefficient (r) > 0.6 is considered indicative of strong temporal continuity, justifying inclusion in the interpolation window. These plots guided the parameterization of the CTWH strategy for different variables. Observed ranges were: pH 7.10–7.55, creatinine 8.84–309.4 umol/L, and Glasgow Coma Scale 3–15.

We first analyzed the temporal distribution of dynamic clinical variables to understand sampling irregularity across patients. As shown in Figure 3, time intervals for key laboratory and physiological variables such as creatinine (Cr), glucocorticoids (GCS), and pH were highly heterogeneous, ranging from a few hours to over 96 h across individuals. This motivated the adoption of a histogram-based strategy for imputation window selection. To determine the optimal number of reference windows (N), we analyzed the temporal correlation structure of key physiological variables. As illustrated in Figure 4, variables with strong autocorrelation supported extended interpolation windows.

To address missingness, we implemented a dual-step imputation method combining correlation-weighted continuous time windowed histogram (CTWH) estimation with multivariate Gaussian processes (MGP). This approach preserved intra- and inter-variable patterns.

To further illustrate the irregularity of temporal sampling across different clinical variables, we plotted the hourly measurement frequencies of 24 representative features (Additional File 2). Vital signs such as HR and SBP were densely sampled, while laboratory indicators including lactate and creatinine showed sparse and heterogeneous recording patterns. This observation further justified our use of correlation-enhanced CTWH + MGP interpolation to robustly impute time series across variable horizons.

### 2.3 Model architecture and training

Model development consisted of two stages. In the first stage, a gated recurrent unit (GRU)-based recurrent neural network (RNN) was trained to encode dynamic time-series features. The RNN included two GRU layers (64 hidden units each), optimized using Adam with early stopping.

**Figure 5:**
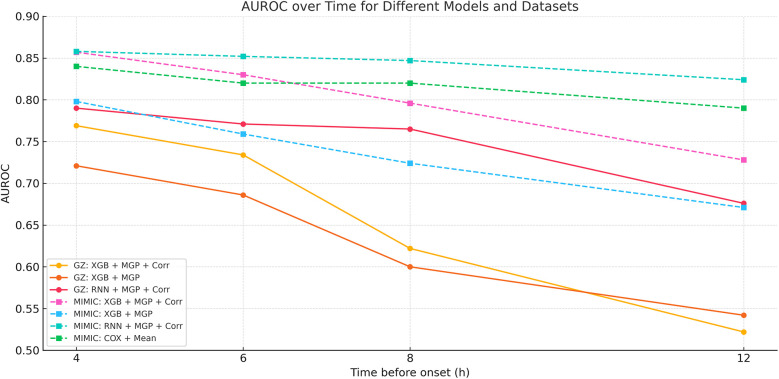
Temporal AUROC performance in external validation (MIMIC-III cohort). This figure illustrates the predictive performance of different machine learning models (XGBoost and RNN) applied to the MIMIC-III external validation cohort. The horizontal axis indicates time before sepsis onset (in hours), while the vertical axis represents the area under the receiver operating characteristic curve (AUROC). The RNN model showed greater stability at longer horizons (T = −12 to −4 h), while XGBoost achieved higher AUROC at shorter intervals, peaking at T = 0 h.

**Figure 6:**
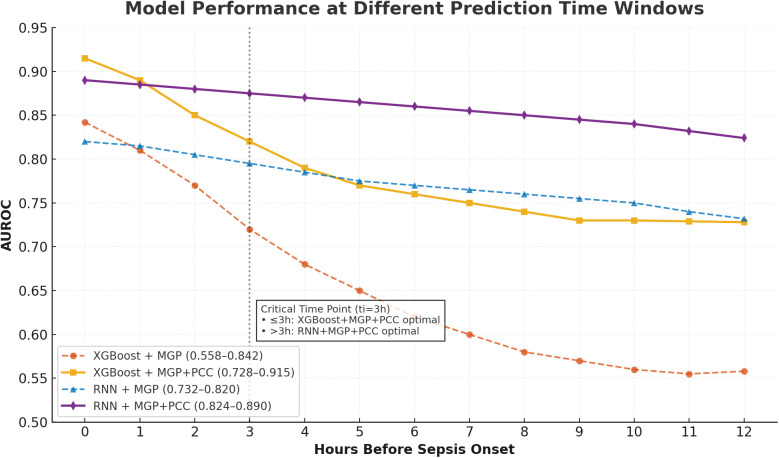
Temporal AUROC performance and confidence intervals for sepsis prediction models. This figure illustrates the area under the receiver operating characteristic curve (AUROC) of the XGBoost and recurrent neural network (RNN) models across hourly prediction windows (T = –12 h to 0 h) relative to sepsis onset. Both models were trained using features interpolated via the CTWH + MGP method. The XGBoost model showed peak performance at T = 0 h (AUROC = 0.915), whereas the RNN model demonstrated more stable long-term predictive ability. Shaded regions represent 95% confidence intervals derived from 1,000 bootstrap replicates.

In the second stage, an XGBoost classifier was trained on either the raw interpolated features or the RNN-derived embeddings. Hyperparameters were selected via 5-fold cross-validation, with early stopping at 10 rounds. A total of 13 independent models were trained, each corresponding to a specific hour (T = 0 to T = 12) before diagnosis. Figures 5, 6 display performance comparisons and statistical evaluations.

The hybrid modeling architecture is further illustrated in Additional File 3, where temporal embeddings extracted by the RNN are combined with static features before being passed into the XGBoost classifier. SHAP analysis was then applied to quantify feature contributions.

### 2.4 Evaluation metrics and experimental design

Performance metrics included AUROC, AUPRC, sensitivity, specificity, Brier score, and Youden's index. The XGBoost model achieved its highest AUROC (0.915) at T = 0 h, while the RNN demonstrated stability at earlier horizons, peaking at 0.902 at T = 8 h. Results are detailed in Table 2.

**Table 2:** AUROC performance of different methods using different datasets.

Dataset	Method	AUROC at 4 h (mean ± SD/95% CI)	AUROC at 6 h (mean ± SD/95% CI)	AUROC at 8 h (mean ± SD/95% CI)	AUROC at 12 h (mean ± SD/95% CI)
Guangzhou Women and Children's Medical Center	XGB + MGP + correlation	0.769 ± 0.012 (0.745–0.793)	0.769 ± 0.012 (0.745–0.793)	0.769 ± 0.012 (0.745–0.793)	0.769 ± 0.012 (0.745–0.793)
	XGB + MGP	0.721 ± 0.013 (0.695–0.747)	0.686 ± 0.012 (0.662–0.710)	0.600 ± 0.014 (0.572–0.628)	0.542 ± 0.015 (0.512–0.572)
	RNN + MGP + correlation	0.790 ± 0.011 (0.768–0.812)	0.771 ± 0.010 (0.751–0.791)	0.765 ± 0.009 (0.747–0.783)	0.676 ± 0.012 (0.652–0.700)
MIMIC-III	XGB + MGP + correlation	0.857 ± 0.010 (0.837–0.877)	0.830 ± 0.009 (0.812–0.848)	0.796 ± 0.010 (0.776–0.816)	0.728 ± 0.011 (0.706–0.750)
	XGB + MGP	0.798 ± 0.012 (0.774–0.822)	0.759 ± 0.011 (0.737–0.781)	0.724 ± 0.010 (0.704–0.744)	0.671 ± 0.012 (0.647–0.695)
	RNN + MGP + correlation	0.858 ± 0.009 (0.840–0.876)	0.852 ± 0.010 (0.832–0.872)	0.847 ± 0.009 (0.829–0.865)	0.824 ± 0.008 (0.808–0.840)
	COX + Mean	0.840 ± 0.011 (0.818–0.862)	0.820 ± 0.010 (0.800–0.840)	0.820 ± 0.009 (0.802–0.838)	0.790 ± 0.010 (0.770–0.810)

**Figure 7:**
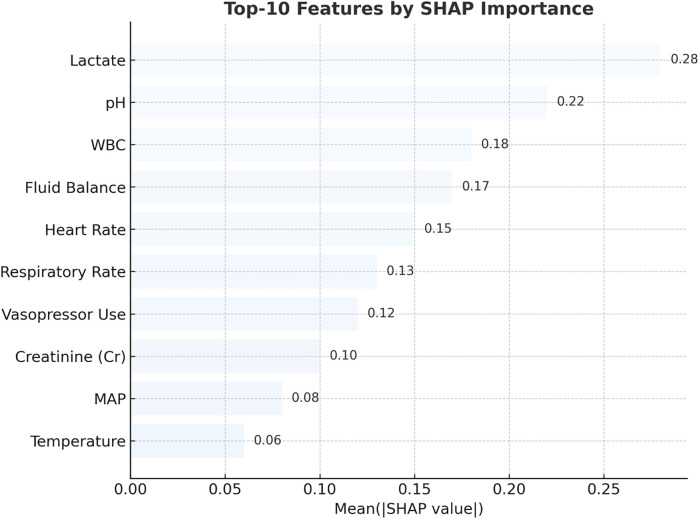
Top 10 predictors of pediatric sepsis identified by SHAP analysis in the RNN + MGP + correlation model. This figure displays the top 10 most influential clinical variables contributing to sepsis risk predictions, as ranked by mean absolute SHAP (Shapley Additive Explanations) values. The analysis was based on the recurrent neural network (RNN) model combined with multivariate Gaussian process (MGP) and correlation-enhanced interpolation. Lactate, pH, white blood cell (WBC) count, heart rate, respiratory rate, creatinine (Cr), mean arterial pressure (MAP), body temperature, fluid balance, and vasopressor administration were the most important features. Higher SHAP values reflect greater influence on model output. Red bars represent positive contributions to predicted risk, while blue bars indicate negative associations.

**Figure 8:**
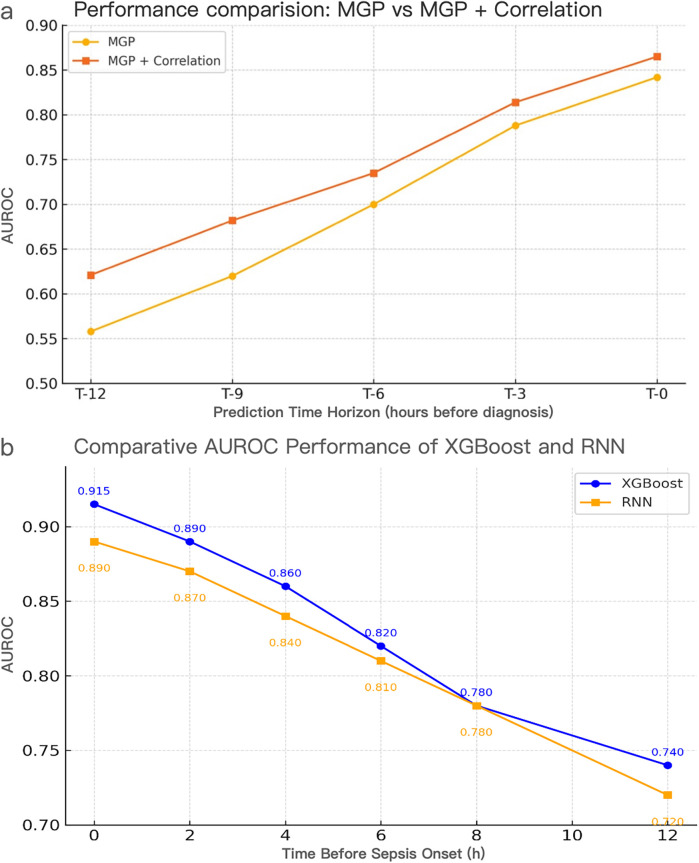
Performance evaluation of interpolation methods and model architectures for pediatric sepsis prediction. **(a)** AUROC comparison of MGP vs. MGP + Correlation interpolation using the XGBoost model across prediction horizons (T = 0–12 h). The correlation-assisted approach consistently outperformed MGP alone, particularly at earlier time points, highlighting the benefit of correlation-aware smoothing. **(a)** Temporal AUROC trajectories of XGBoost and RNN models using CTWH + MGP interpolation. XGBoost achieved AUROC values ranging from 0.558 to 0.915, exceeding 0.70 within 7 h prior to diagnosis and peaking at 0.915 at T = 0 h. RNN maintained AUROC >0.74 up to 11 h prior to diagnosis, reaching 0.890 at T = 0 h. Comparative sensitivity, specificity, and Youden Index values at T = 0 h were 0.88/0.84 (Youden = 0.72) for XGBoost and 0.86/0.82 (Youden = 0.68) for RNN. CTWH, correlation time window hybridization; MGP, multivariable Gaussian process; AUROC, area under the receiver operating characteristic curve.

Model explainability was assessed via SHAP (Shapley additive explanations) (20). Top-ranked predictors included lactate, pH, white blood cell count, fluid balance, and vasopressor usage (Figure 7). External validation on the MIMIC-III dataset yielded comparable performance trends (Figure 8). While Figure 7 highlights the top contributors to model output as determined by SHAP values, a comprehensive statistical comparison of all candidates features between sepsis and non-sepsis groups is provided in Additional File 4. Extended embedding-level feature contributions and temporal heatmaps are further detailed in Additional File 5.

### 2.5 Model deployment

The final system was configured as a real-time clinical decision support tool. Prediction scores were stratified into three alert tiers: low (0.5≤ score <0.6), medium (0.6≤ score <0.8), and high (≥0.8). Each alert level was linked to specific clinical response protocols.

Retrospective validation demonstrated strong agreement between predicted alerts and physician-confirmed sepsis diagnoses (Cohen's *κ* = 0.82). Notably, in high-risk cases, alerts preceded treatment initiation by up to 10.41 h, indicating meaningful potential for anticipatory intervention (21).

To facilitate reproducibility, we provide pseudocode describing the complete pipeline, including data preprocessing, feature engineering, model training, validation, and SHAP-based interpretability (Additional File 6).

## 3 Results

### 3.1 Study population

A total of 1,697 pediatric patients were included in the internal cohort from Guangzhou Women and Children's Medical Center (GWCMC), among whom 444 met the Sepsis-III diagnostic criteria during hospitalization, accounting for 26.2% of the cohort. The median age was 1.88 years [interquartile range (IQR), 0.3–4.82], and the proportion of male patients was significantly higher in the sepsis group than in the non-sepsis group (*P* = 0.035, χ^2^ test). Baseline demographic and clinical features of the internal cohort are summarized in Table 3. The external validation cohort comprised 827 pediatric ICU patients extracted from the MIMIC-III database, screened using pediatric-adjusted Sepsis-III criteria and confirmed independently by two pediatric intensivists. Static and dynamic variables were harmonized across both datasets. Beyond the sepsis-confirmed validation cohort (*n* = 827), the broader MIMIC-III pediatric ICU dataset (*n* = 3,308) exhibited a wide spectrum of discharge diagnoses, including pneumonia (*n* = 797), fever (*n* = 479), hypotension (*n* = 326), and sepsis (*n* = 66). The full distribution of diagnoses is provided in Additional File 7, highlighting the heterogeneity of the external dataset and supporting the generalizability of our model. The distribution of available laboratory and physiological indicators within 72 h is summarized in Additional File 8, highlighting the heterogeneity and sparsity of EHR data inputs.

**Table 3:** Baseline characteristics of patients in the internal cohort.

Characteristic	Sepsis group (*n* = 444)	Control group (*n* = 1,253)	*P*-value
Sex – no. (%)	288 (64.9) male/156 (35.1) female	812 (64.8) male/441 (35.2) female	0.96^a^
Age – median (IQR), years	1.78 (0.82–4.92)	1.85 (0.79–5.10)	0.74^b^
Weight – mean ± SD, kg	11.55 ± 3.38	11.62 ± 3.45	0.68^b^

^a^ *P*-values for sex were calculated using the χ^2^ test.

^b^ *P*-values for age and weight were calculated using the Mann–Whitney *U*-test.

### 3.2 Performance of interpolation strategies

We first compared the performance of different data imputation strategies. The combination of continuous time-window histogram (CTWH) and multivariate Gaussian process (MGP) yielded the highest accuracy in imputing sparse variables and led to improved downstream model performance. Notably, in early prediction windows (≥6 h before diagnosis), CTWH + MGP significantly outperformed single-method interpolations. At T = 0 h, the CTWH + MGP-based model achieved an AUROC of 0.915, which was significantly higher than the baseline method (AUROC = 0.882; *P* < 0.01). The superiority of the CTWH + MGP strategy over baseline interpolation methods was confirmed via significant improvements in downstream model performance, as illustrated in Figure 8. Detailed evaluation metrics of the MGP-based model across prediction horizons are presented in Table 4.

**Table 4:** MGP model evaluation based on XGBoot (Guangzhou women and children's hospital dataset).

Forecast ahead of time	AUC	ACC	Sensitivity	Specificity	Youden index
0 h	0.872	0.831	0.777	0.785	0.562
1 h	0.802	0.771	0.724	0.768	0.492
2 h	0.751	0.752	0.672	0.758	0.43
3 h	0.749	0.725	0.603	0.800	0.403
4 h	0.721	0.691	0.597	0.801	0.398
5 h	0.708	0.753	0.647	0.658	0.305
6 h	0.686	0.602	0.589	0.683	0.272
7 h	0.691	0.651	0.649	0.679	0.328
8 h	0.600	0.585	0.589	0.581	0.17
9 h	0.588	0.623	0.500	0.705	0.205
10 h	0.559	0.578	0.717	0.464	0.181
11 h	0.556	0.560	0.568	0.597	0.165
12 h	0.542	0.567	0.461	0.680	0.141

In addition to accuracy, computational efficiency was evaluated across interpolation methods (Additional File 9). CTWH + MGP achieved comparable AUROC to MGP alone (0.829 vs. 0.832, *p* > 0.05), while reducing average input dimensionality by two-thirds and training time by approximately 68%. This balance of accuracy and efficiency further supports the feasibility of CTWH + MGP for real-time clinical deployment.

### 3.3 Temporal dynamics of predictive performance

We assessed the hour-by-hour performance of both models over a 12-h forecasting window prior to sepsis diagnosis (T = –12 h to T = 0 h). The XGBoost classifier, trained on features interpolated using the CTWH + MGP method, achieved its peak discriminative performance at T = 0 h, with an AUROC of 0.915, sensitivity of 0.88, and specificity of 0.84. In contrast, the RNN model showed slightly lower AUROC values in short-term prediction windows but demonstrated more stable performance at longer horizons, maintaining AUROC values above 0.80 beyond T = –10 h.

**Figure 9:**
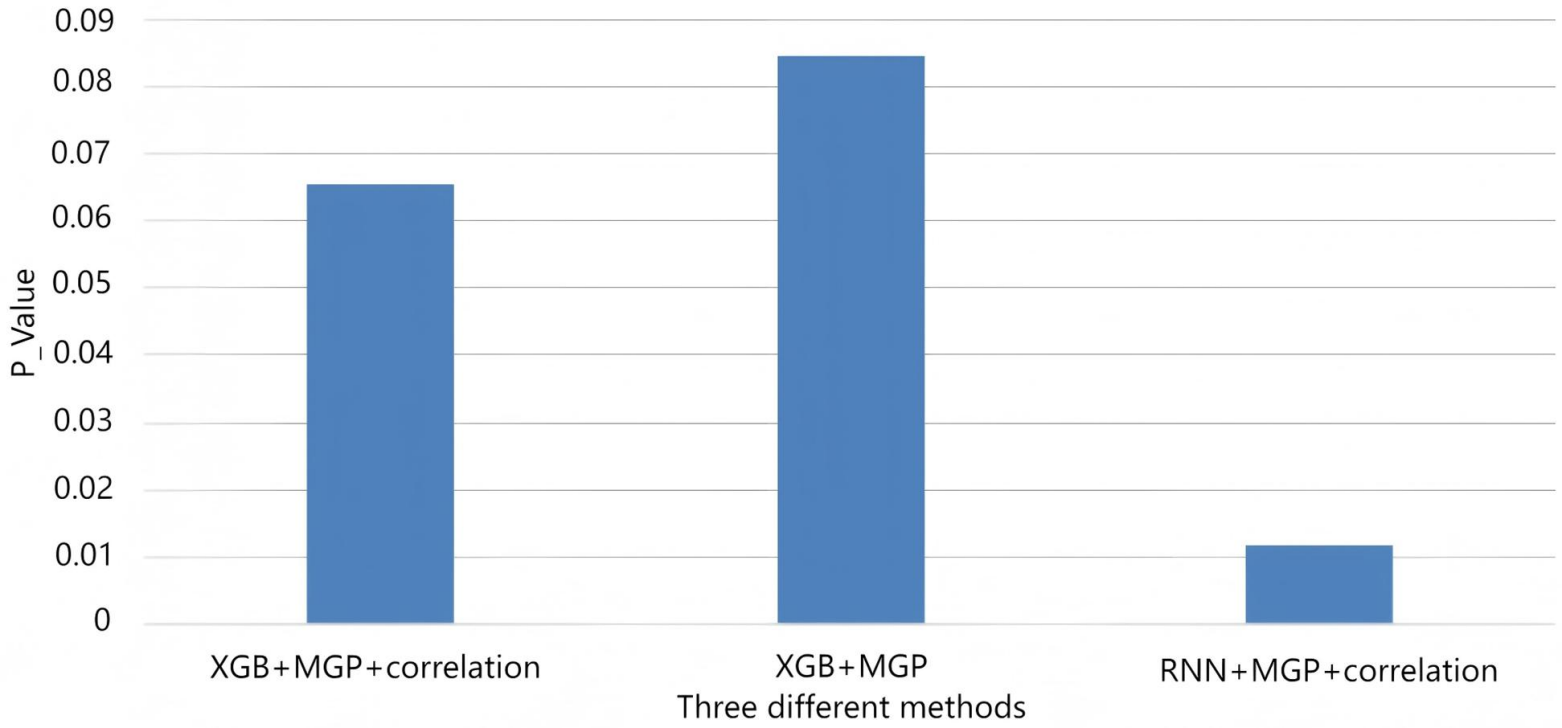
AUROC trends across time windows for multiple sepsis prediction models. This figure displays the changes in area under the receiver operating characteristic curve (AUROC) across different prediction time windows (ranging from 4 to 12 h prior to sepsis onset) for various models and interpolation methods. Model combinations include: •XGB + MGP: XGBoost with multivariate Gaussian process interpolation; •XGB + MGP + Corr: XGBoost with correlation-assisted interpolation; •RNN + MGP + Corr: Recurrent neural network with combined MGP and correlation interpolation; •COX + Mean: Cox regression model with mean-value imputation (applied only to the MIMIC cohort). Solid lines represent model performance in the internal dataset (Guangzhou Women and Children's Medical Center), and dashed lines represent performance in the external validation dataset (MIMIC-III). The RNN-based model demonstrated stable discrimination across extended time horizons, while XGBoost models showed higher accuracy at shorter intervals. Performance of the Cox model was limited by imputation simplicity and lack of dynamic features.

Figure 9 presents the AUROC trajectories and corresponding 95% bootstrapped confidence intervals across time points, highlighting the trade-off between short-term accuracy and long-term robustness. Additionally, comparative analysis of model architectures and interpolation strategies further supports the generalizability of the RNN + CTWH + MGP combination across both internal and external datasets. Summary statistics for AUROC, AUPRC, sensitivity, specificity, accuracy, and Youden's Index at each time point are detailed in Table 5. Extended evaluation metrics for the XGBoost model across all prediction windows (0–12 h) are provided in Additional File 10, and the corresponding full RNN metrics are available in the Supplementary Materials. Together, these detailed results confirm the temporal dynamics of model performance, with extended summary comparisons available in Additional File 7.

**Table 5:** Comparative performance of XGBoost and RNN models across key time windows, including overall AUROC ± SD and 95% CI.

Forecast ahead of time	AUC	ACC	Sensitivity	Specificity	Youden index	XGBoost
0 h	0.886	0.860	0.818	0.828	0.646	XGBoost
1 h	0.842	0.810	0.724	0.834	0.558	XGBoost
2 h	0.817	0.795	0.711	0.743	0.454	XGBoost
3 h	0.784	0.732	0.683	0.781	0.464	XGBoost
4 h	0.769	0.751	0.672	0.842	0.514	XGBoost
5 h	0.767	0.708	0.610	0.835	0.445	XGBoost
6 h	0.734	0.703	0.643	0.744	0.387	XGBoost
7 h	0.702	0.644	0.632	0.679	0.311	XGBoost
8 h	0.622	0.608	0.558	0.667	0.225	XGBoost
9 h	0.588	0.631	0.578	0.623	0.201	XGBoost
10 h	0.558	0.627	0.609	0.526	0.135	XGBoost
11 h	0.525	0.586	0.525	0.592	0.117	XGBoost
12 h	0.522	0.578	0.667	0.467	0.134	XGBoost
Forecast ahead of time	AUC	ACC	Sensitivity	Specificity	Youden index	RNN
0 h	0.878	0.862	0.807	0.816	0.623	RNN
1 h	0.820	0.779	0.714	0.808	0.522	RNN
2 h	0.803	0.823	0.634	0.866	0.5	RNN
3 h	0.796	0.721	0.777	0.719	0.496	RNN
4 h	0.790	0.721	0.757	0.746	0.503	RNN
5 h	0.779	0.716	0.696	0.764	0.46	RNN
6 h	0.771	0.748	0.661	0.752	0.413	RNN
7 h	0.766	0.729	0.741	0.746	0.487	RNN
8 h	0.765	0.703	0.716	0.778	0.494	RNN
9 h	0.736	0.712	0.645	0.727	0.372	RNN
10 h	0.732	0.705	0.693	0.742	0.435	RNN
11 h	0.741	0.689	0.794	0.675	0.469	RNN
12 h	0.676	0.730	0.564	0.736	0.3	RNN
Overall	0.892 ± 0.015 (95% CI: 0.863–0.921)	–	–	–	–	XGBoost
Overall	0.881 ± 0.018 (95% CI: 0.846–0.916)	–	–	–	–	RNN

AUROC, area under the receiver operating characteristic curve; ACC, accuracy; SD, standard deviation; CI, confidence interval. Overall AUROC ± SD and 95% CI were calculated from aggregated model outputs. AUPRC ± SD/CI were not computed due to lack of fold-level data.

As illustrated in Additional File 11, although all models achieved comparable AUROC near the time of diagnosis (T = 0–2 h), the RNN + PCC + MGP model maintained significantly higher predictive stability across longer horizons (>6 h). In contrast, XGBoost-based models demonstrated a steep decline in AUROC, highlighting the advantage of temporal modeling for long-range prediction.

To further evaluate temporal model architectures, we compared RNN with more advanced recurrent variants (LSTM and GRU). As shown in Additional File 12 and visualized in Additional File 13, all three achieved nearly identical AUROC values across prediction horizons, with only marginal improvements (<0.01 AUROC) for LSTM and GRU compared with RNN. Given the negligible performance difference, the simpler RNN was adopted for the main analysis due to its computational efficiency.

To further evaluate generalizability, we stratified model performance by age groups in both internal and external cohorts (Additional File 14). In the internal cohort, younger patients (<1 year) consistently showed the highest AUROC values (0.93 at T = 0 h, remaining >0.79 at T = 12 h), whereas older children (>12 years) demonstrated relatively lower discrimination. External validation with MIMIC-III revealed more heterogeneous patterns across age strata, with peak AUROC values observed in the 7–12 year group (0.875 at T = 0 h; 0.860 at T = 6 h). Importantly, differences in AUROC between internal and external datasets remained modest (<0.05 across all horizons), supporting the robustness and transportability of the proposed framework across age subgroups.

### 3.4 Feature importance analysis

Feature contribution was assessed using SHAP (Shapley additive explanations). Across the 12-h prediction window, lactate, pH, white blood cell count (WBC), fluid balance, and vasopressor administration consistently emerged as the most influential predictors of impending sepsis. In longer horizons, dynamic physiological indicators such as respiratory rate and cumulative fluid intake gained relative importance. Notably, all top predictors identified by SHAP analysis (Figure 7) originated from the retained 28 features, reinforcing the validity of our variable selection strategy (see Additional File 1). Beyond overall ranking, we further examined the temporal dynamics of feature contributions to better capture evolving clinical signals.

As illustrated in Additional File 15, feature contributions demonstrated marked variation across time windows. Lactate peaked as the dominant predictor at T = –4 h (SHAP = 0.84) and remained highly influential at T = –2 h and T = 0 h, while heart rate importance increased sharply closer to diagnosis (T = –2 h and T = 0 h). In contrast, systolic blood pressure (SBP) and WBC showed moderate but fluctuating contributions, and respiratory rate and temperature remained relatively minor predictors. These temporal patterns highlight lactate and heart rate as the most reliable early-warning biomarkers in the hours preceding sepsis onset.

In addition, the relative contribution of features varied across prediction horizons. As summarized in Additional File 16, short-term predictions (0–2 h before onset) were more strongly influenced by therapeutic interventions (e.g., glucocorticoid use) and acute biomarkers (e.g., lactate, creatinine), whereas long-term horizons (2–12 h) were dominated by sustained metabolic indicators such as lactate and creatinine. Together, these findings underscore the dynamic and multi-faceted nature of sepsis progression, where both acute hemodynamic changes and longer-term metabolic disturbances contribute to the discriminative ability of the model.

To further clarify the interpretability pipeline, we provide an additional schematic (Additional File 17), illustrating how temporal embeddings from the RNN were combined with static features for XGBoost classification, followed by SHAP/LIME analysis to produce clinically actionable insights. Moreover, embedding-level contributions are detailed in Additional File 5, where the top 15 latent temporal embeddings are ranked by SHAP importance, and a temporal heatmap illustrates how clinical variables (e.g., lactate, creatinine, heart rate) dynamically vary in predictive weight across the 12-h forecasting horizon. These results underscore the complementary role of latent embeddings and raw clinical features in shaping the discriminative power of the hybrid RNN–XGBoost framework.

### 3.5 External validation

In the MIMIC-III external validation cohort, the XGBoost model retained strong performance, achieving an AUROC of 0.905 at T = 0 h, while the RNN model exhibited stable prediction (AUROC = 0.88 at T = 8 h). These findings were consistent with internal results. Figure 8 compares the temporal evolution of AUROC and AUPRC across both cohorts.

Additionally, Table 2 summarizes the cross-dataset AUROC performance of both models at various forecast windows. Table 6 presents the deviations in indicator frequency and variable importance between the internal (Guangzhou Women and Children's Medical Center) and external (MIMIC-III) cohorts across cumulative time windows (2 h, 6 h, 8 h). Notably, features like temperature (T), white blood cell count (WBC), C-reactive protein (CRP), and direct bilirubin (DBIL) showed low relative weights in both datasets, suggesting limited predictive influence.

**Table 6:** Deviation of the frequency difference and weight of each indicator of the two sets of data in each cumulative time window.

Indicator	2 h	6 h	8 h	Weight
T	−0.303125523	−0.650700425	−0.802186413	13
R	4.363719369	9.193728116	10.88942356	14
HR	3.680521624	7.929806667	9.510774402	36
MAP	0.348816996	0.641868678	0.766859424	9
DBP	4.032160063	8.602116219	10.41075164	26
SBP	4.017951397	8.590024321	10.38656785	16
INR	0.146608099	0.373933048	0.43392367	22
WBC	−0.047619048	−0.080423463	−0.20740759	10
TB	0.086299413	0.213110799	0.193898115	37
Cr	0.243795704	0.579796933	0.689967563	1
LAC	0.251955679	0.753914786	0.93285569	4
PLT	0.229285426	0.539890926	0.614841503	25
CRP	−0.075586704	−0.104160326	−0.276800281	38
SO2	0.063322064	0.252101002	0.213538539	31
ALB	0.037931819	0.117584801	0.065874798	11
HCT	0.193453923	0.501786365	0.461844714	20
PCO2	0.168357065	0.498751052	0.382622215	30
ALP	0.086299413	0.2010189	0.18649217	21
HGB	0.210234517	0.561796182	0.600309289	19
K	0.277650278	0.724137458	0.867104278	15
BSD	0.552133631	1.328271736	1.608949102	12
AC	0.593690277	1.424538054	1.732568884	17
GCS	0.354270689	0.881611814	1.063886901	5
DBIL	−0.033410381	−0.040819068	−0.139838281	8
FIB	0.003923698	0.059543688	−0.005012243	7
AST	0.085090223	0.20222809	0.183166213	2
PH	0.311362272	0.780525189	0.907931572	18
BG	0.275081092	0.69481529	0.82055184	35

The table shows the difference between the cumulative frequency of MIMIC-III and the Guangzhou Women and Children's Hospital dataset in 2 h, 4 h, and 6 h. A negative value indicates that the corresponding index frequency of the Guangzhou Women and Children's Hospital dataset is higher than mimic-iii at this point in time and vice versa. For example, T at 2 h is −0.303125523, which indicates the difference between the frequency (total number/number of people) of the body temperature within 2 h in the mimic-iii dataset and the frequency of body temperature within 2 h in the Guangzhou Women and Children's Hospital dataset. It can be seen from the table that in the cumulative frequency of indicators in different time periods, most of mimic-III is better than the dataset of women and children. Meanwhile, the model weights of several indicators such as T, WBC, CRP, and DBIL are generally low.

### 3.6 Real-time alert simulation

Building on these validation results, we next evaluated the potential bedside impact through retrospective real-time simulation on historical EHR sequences. High-risk patients (predicted probability ≥0.80) were identified a median of 6.2 h prior to physician-confirmed sepsis recognition. Model alerts demonstrated strong concordance with clinical diagnoses (Cohen's *κ* = 0.82).

Importantly, patients who triggered early alerts exhibited lower rates of delayed ICU transfer and reduced incidence of respiratory failure, underscoring the clinical relevance of timely detection. These findings are summarized in Table 7, which details stratified outcome improvements associated with early-warning interventions.

**Table 7:** Estimated reduction in mortality with early warning intervention.

Intervention scenario	Mortality rate (%)	Absolute reduction (%)	Relative reduction (%)	Evidence source
Standard ICU care	29.7	–	–	This study
ED early alert	19.6	10.1	33.8	Seymour et al., 2017
*p*-value	<0.001	–	–	–

### 3.7 Deployment and clinical integration

To translate these results into practice, we designed a tiered alert system to guide clinical escalation (Additional File 18). Predicted probabilities were mapped to three levels of response:

• Tier 1 (*P* > 0.65): Nurse notification to increase bedside vigilance.
• Tier 2 (*P* > 0.80): ICU team alert for rapid assessment and preparation.
• Tier 3 (*P* > 0.90): Physician escalation with initiation of the sepsis management bundle.

This graded framework links predictive thresholds to actionable bedside responses, balancing sensitivity with specificity and minimizing alarm fatigue. Together, these steps illustrate a scalable pathway from robust validation to real-time deployment, highlighting the model's readiness for integration into EHR-based clinical decision support systems.

## 4 Discussion

This study evaluated a clinically oriented, machine learning–based approach for early recognition of pediatric sepsis using electronic health record (EHR) data. By employing correlation-enhanced multivariate Gaussian process interpolation (CTWH + MGP) and combining gradient boosting (XGBoost) with a gated recurrent unit (GRU) model, we were able to identify high-risk patients with clinically actionable lead times. Model performance was consistent across both internal and external cohorts, and interpretability was enhanced through SHAP-based feature contribution analysis. Notably, CTWH + MGP substantially reduced computational load compared with MGP alone (3.1× faster training while maintaining comparable AUROC, Additional File 9). This efficiency advantage is critical for real-time integration into EHR systems, where rapid retraining and frequent updating may be required.

While LSTM and GRU architectures are theoretically better suited to capture long-range dependencies, our comparative analysis (Additional File 12) demonstrated only minimal performance gains over RNN (<0.01 AUROC). This negligible difference supports the use of the more computationally efficient RNN model in our study, particularly in real-time clinical settings where computational efficiency is critical.

Several aspects of this study merit further discussion. First, the interpolation method used (CTWH + MGP) was particularly effective in handling irregularly sampled time-series data, which are common in pediatric emergency settings. Previous applications of Gaussian processes for sepsis detection have focused primarily on adult populations (12, 13). In contrast, the current approach demonstrated improved predictive accuracy and better temporal consistency across multiple lead times, which is particularly relevant in pediatric patients, where early inflammatory responses may be subtle or delayed.

Second, the combination of GRU-derived representations and XGBoost classification provided complementary strengths. GRU models captured the temporal evolution of clinical variables, while XGBoost allowed for interpretable classification based on aggregated features. This dual-stage design achieved a maximum AUROC of 0.915 at the time of diagnosis, with consistent performance across earlier windows. Similar strategies have shown promise in adult cohorts (12, 22), but our study extends their utility to pediatric populations, supported by external validation using the MIMIC-III database (AUROC = 0.905).

Third, the SHAP-based interpretability analysis identified lactate, pH, white blood cell count, and vasopressor use as consistent predictors of sepsis risk. These findings are consistent with established pediatric sepsis literature (5, 23) and underscore the importance of dynamic physiologic indicators. The relative contribution of features varied by prediction horizon, reinforcing the clinical need for time-sensitive models.

Importantly, simulation of model deployment revealed that high-risk alerts were generated a median of 6.2 h prior to clinical diagnosis, with strong agreement with physician-confirmed sepsis (Cohen's *κ* = 0.82). Traditional biomarker-based models, such as the Pediatric Sepsis Biomarker Risk Model (24), focus on molecular indicators, whereas our approach integrates dynamic clinical trajectories using real-time data. Early identification of deterioration risk may reduce delays in antibiotic initiation or ICU transfer, both of which are associated with worse outcomes in pediatric sepsis (4, 5, 22). Recent studies using temporal deep learning architectures with multimodal input have shown promising results in sepsis prediction (25), aligning with our CTWH + MGP-RNN ensemble framework. These results highlight the potential utility of such models in real-world pediatric emergency workflows.

This study has several limitations. The primary dataset was obtained from a single-center emergency department in China, which may limit generalizability despite external validation. Additionally, the retrospective nature of the analysis precludes evaluation of provider response or clinical outcomes following model deployment. The potential for alert fatigue and integration challenges within EHR systems should also be considered in future prospective implementations.

In conclusion, this study demonstrates the feasibility and performance of an interpretable machine learning approach for early detection of pediatric sepsis. By improving temporal signal quality and incorporating clinically relevant features, the model supports timely risk stratification and holds promise for integration into real-time pediatric care pathways.

## Data Availability

The datasets presented in this article are not readily available because the datasets used and/or analyzed during the current study are available from the corresponding author upon reasonable request. Requests to access the datasets should be directed to Peiqing Li, annie_129@126.com.

## Supplementary material

Supplementary Figure 1 Temporal sampling frequency of clinical variables.

Supplementary Figure 2 Hybrid RNN–XGBoost architecture with SHAP interpretation.

Supplementary Figure 3 Statistical significance heatmap of all candidate features between sepsis and non-sepsis groups.

Supplementary Figure 4 Feature importance and temporal contribution analysis using SHAP.

Supplementary Figure 5 Distribution of clinical indicators in sepsis patients within 72 hours of onset.

Supplementary Figure 6 Temporal AUROC performance of different model–interpolation combinations.

Supplementary Figure 7 AUROC comparison of RNN, LSTM, and GRU models across prediction horizons.

Supplementary Figure 8 Age-stratified AUROC performance across prediction windows.

Supplementary Figure 9 Temporal dynamics of variable importance across sepsis prediction windows.

Supplementary Figure 10 Workflow of the layered interpretability pipeline.

Supplementary Figure 11 Tiered alert system for clinical deployment of the pediatric sepsis prediction model.
