# Supplementary material for: Accurate prediction of sepsis from pediatric emergency department to PICU using a machine-learning model
Source: Front Pediatr. 2025 Oct 10;13:1610187. doi: 10.3389/fped.2025.1610187 (PMC12550503; doi:10.3389/fped.2025.1610187)
Supplement: Supplementary file 1 [file Supplementaryfile1.docx]

**Additional File 1.** Included and excluded clinical indicators based on a 20% missingness threshold in the model dataset.

| Category​**​** | Included (n=28)​**​** | Excluded (n=16)​**​** | Missing Rate​**​** | Selection Rationale​ |
| --- | --- | --- | --- | --- |
| Vital Signs | HR、RR、SpO₂、T | Arterial blood pressure | <10% | Guide Recommended Core Parameters |
|  |  |  | 28%↑ | >20%Missing thresholds |
| Laboratory Indicators​​ | WBC、Cr、Lac、pH | PCT | <15% | Key Biomarkers in Sepsis |
|  |  |  | 42%↑ | Insufficient availability |
| Medicine​ | Boosters 、antibiotics、GCS | - | 12% | Treatment-related |
| Fluid balance​​ | Urine output and fluid intake | - | 8% | Organ perfusion indicators |
| Exclude metrics (n=16) | - | Bilirubin, Troponin、IL-6、PaCO₂ | 25-50% | >20%Missing Rate |

**Note:** Vital signs, laboratory indicators, medication data, and fluid balance metrics were assessed. Indicators with >20% missing data were excluded. MGP = Multivariate Gaussian Process; HR = Heart Rate; RR = Respiratory Rate; SpO₂ = Oxygen Saturation; T = Temperature; WBC = White Blood Cell count; Cr = Creatinine; Lac = Lactate; GCS = Glasgow Coma Scale.
