# Supplementary material for: Accurate prediction of sepsis from pediatric emergency department to PICU using a machine-learning model
Source: Front Pediatr. 2025 Oct 10;13:1610187. doi: 10.3389/fped.2025.1610187 (PMC12550503; doi:10.3389/fped.2025.1610187)
Supplement: Supplementary file 2 [file Supplementaryfile2.docx]

Additiongal File 6. Pseudocode for Model Development and Evaluation

**Code**

# Step 1: Load libraries and data

import pandas as pd, numpy as np

from sklearn.model_selection import StratifiedKFold

from sklearn.preprocessing import StandardScaler, OneHotEncoder

from xgboost import XGBClassifier

import torch

import torch.nn as nn

# Step 2: Data preprocessing

# - Remove physiologically implausible values

# - Drop features with >20% missingness

# - Impute using time-window + correlation, fallback to MGP

# - Standardize continuous variables (z-score)

# - One-hot encode categorical variables

# - Derive temporal features (recent value, mean, max, slope)

# Step 3: Define models

# XGBoost hyperparameters: tuned via TPE

xgb = XGBClassifier(

n_estimators=300, max_depth=4, learning_rate=0.05,

subsample=0.8, colsample_bytree=0.8, min_child_weight=2,

reg_lambda=1.0, scale_pos_weight=pos_weight

)

# RNN architecture: 2-layer GRU, hidden_size=128, dropout=0.2

class GRUModel(nn.Module):

def __init__(self):

super().__init__()

self.gru = nn.GRU(input_size=num_features, hidden_size=128,

num_layers=2, dropout=0.2, batch_first=True)

self.fc = nn.Linear(128, 1)

def forward(self, x):

out, _ = self.gru(x)

out = out[:, -1, :]

return torch.sigmoid(self.fc(out))

# Step 4: Cross-validation

kf = StratifiedKFold(n_splits=10, shuffle=True, random_state=42)

for train_idx, test_idx in kf.split(X, y):

# Split data

# Train model (XGBoost or RNN)

# Evaluate: AUROC, AUPRC, Sensitivity, Specificity, Calibration

# Step 5: External validation

# - Apply trained model to MIMIC-III cohort

# - Report same metrics

# Step 6: Model interpretability

# - Compute SHAP values for top features

# - Generate summary plot and feature importance chart
