# Supplementary material for: Accurate prediction of sepsis from pediatric emergency department to PICU using a machine-learning model
Source: Front Pediatr. 2025 Oct 10;13:1610187. doi: 10.3389/fped.2025.1610187 (PMC12550503; doi:10.3389/fped.2025.1610187)
Supplement: Supplementary file 3 [file Supplementaryfile3.docx]

**Additional File 7.** Patients from the MIMIC-III dataset

| **Discharge_Diag_Name** | **Sex_Name** | | **counts** |
| --- | --- | --- | --- |
|  | **female** | **male** |  |
| **MITRAL REGURGITATION** | 1 | 5 | 6 |
| **ABDOMINAL PAIN** | 318 | 402 | 720 |
| **ABSCESS** | 2 | 4 | 6 |
| **ACUTE CHOLANGITIS** | **2** | **4** | **6** |
| **ACUTE CHOLECYSTITIS** | **1** | **5** | **6** |
| **ACUTE PULMONARY EMBOLISM** | **5** | **2** | **7** |
| **ACUTE RESPIRATORY DISTRESS SYNDROME;ACUTE RENAL FAILURE** | **5** | **2** | **7** |
| **ACUTE SUBDURAL HEMATOMA** | **4** | **3** | **7** |
| **ALCOHOLIC HEPATITIS** | **4** | **2** | **6** |
| **ALTERED MENTAL STATUS** | **3** | **4** | **7** |
| **AROMEGLEY;BURKITTS LYMPHOMA** | **1** | **5** | **6** |
| **ASTHMA/COPD FLARE** | **1** | **6** | **7** |
| **ASTHMA;CHRONIC OBST PULM DISEASE** | **8** | **4** | **12** |
| **BASAL GANGLIN BLEED** | **2** | **4** | **6** |
| **BRADYCARDIA** | **5** | **2** | **7** |
| **BRAIN METASTASES** | **1** | **5** | **6** |
| **CELLULITIS** | **4** | **2** | **6** |
| **CEREBROVASCULAR ACCIDENT** | **4** | **3** | **7** |
| **CHEST PAIN** | **6** | **1** | **7** |
| **CHEST PAIN/ CATH** | **4** | **2** | **6** |
| **CHOLANGITIS** | **5** | **2** | **7** |
| **CHOLECYSTITIS** | **3** | **3** | **6** |
| **CHRONIC MYELOGENOUS LEUKEMIA;TRANSFUSION REACTION** | **2** | **4** | **6** |
| **CONGESTIVE HEART FAILURE** | **7** | **11** | **18** |
| **CORONARY ARTERY DISEASE\CORONARY ARTERY BYPASS GRAFT /SDA** | **3** | **4** | **7** |
| **CRITICAL AORTIC STENOSIS/HYPOTENSION** | **4** | **2** | **6** |
| **ELEVATED LIVER FUNCTIONS;S/P LIVER TRANSPLANT** | **2** | **4** | **6** |
| **ESOPHAGEAL CA/SDA** | **6** | **8** | **14** |
| **ESOPHAGEAL CANCER/SDA** | **4** | **2** | **6** |
| **FACIAL NUMBNESS** | **2** | **4** | **6** |
| **FAILURE TO THRIVE** | **10** | **10** | **20** |
| **FEVER** | **264** | **215** | **479** |
| **FEVER;URINARY TRACT INFECTION** | **1** | **5** | **6** |
| **GASTROINTESTINAL BLEED** | **4** | **8** | **12** |
| **HEADACHE** | **2** | **4** | **6** |
| **HEPATIC ENCEP** | **2** | **5** | **7** |
| **HEPATITIS B** | **4** | **2** | **6** |
| **HUMERAL FRACTURE** | **1** | **5** | **6** |
| **HYPOGLYCEMIA** | **3** | **4** | **7** |
| **HYPONATREMIA;URINARY TRACT INFECTION** | **2** | **4** | **6** |
| **HYPOTENSION** | **125** | **201** | **326** |
| **HYPOTENSION, RENAL FAILURE** | **2** | **5** | **7** |
| **HYPOTENSION;TELEMETRY** | **1** | **5** | **6** |
| **HYPOTENSION;UNRESPONSIVE** | **3** | **4** | **7** |
| **INFERIOR MYOCARDIAL INFARCTION\CATH** | **2** | **5** | **7** |
| **LEFT HIP FRACTURE** | **4** | **2** | **6** |
| **LEFT HIP OA/SDA** | **3** | **3** | **6** |
| **LIVER FAILURE** | **6** | **6** | **12** |
| **LOWER GI BLEED** | **1** | **5** | **6** |
| **LUNG CANCER;SHORTNESS OF BREATH** | **2** | **4** | **6** |
| **MEDIASTINAL ADENOPATHY** | **3** | **3** | **6** |
| **METASTATIC MELANOMA;BRAIN METASTASIS** | **3** | **3** | **6** |
| **METASTIC MELANOMA;ANEMIA** | **3** | **4** | **7** |
| **MI CHF** | **2** | **5** | **7** |
| **NON SMALL CELL CANCER;HYPOXIA** | **4** | **2** | **6** |
| **OVERDOSE** | **3** | **3** | **6** |
| **PERICARDIAL EFFUSION** | **4** | **3** | **7** |
| **PLEURAL EFFUSION** | **3** | **4** | **7** |
| **PNEUMONIA** | **312** | **485** | **797** |
| **PNEUMONIA/HYPOGLCEMIA/SYNCOPE** | **6** | **1** | **7** |
| **PNEUMONIA;TELEMETRY** | **3** | **4** | **7** |
| **PULMONARY EDEMA, MI** |  | **6** | **6** |
| **PULMONARY EDEMA\CATH** | **2** | **4** | **6** |
| **RECURRENT LEFT CAROTID STENOSIS,PRE HYDRATION** | **5** | **1** | **6** |
| **RENAL CANCER/SDA** | **2** | **5** | **7** |
| **RENAL FAILIURE-SYNCOPE-HYPERKALEMIA** | **3** | **3** | **6** |
| **RESPIRATORY DISTRESS** | **4** | **9** | **13** |
| **RIGHT HUMEROUS FRACTURE** | **1** | **5** | **6** |
| **S/P FALL** | **4** | **3** | **7** |
| **S/P MOTOR VEHICLE ACCIDENT** |  | **6** | **6** |
| **S/P MOTORCYCLE ACCIDENT** | **3** | **3** | **6** |
| **SEIZURE** | **3** | **3** | **6** |
| **SEIZURE;STATUS EPILEPTICUS** | **5** | **2** | **7** |
| **SEPSIS** | **20** | **46** | **66** |
| **UTI/PYELONEPHRITIS** |  | **7** | **7** |
| **SEPSIS;PNEUMONIA;TELEMETRY** |  | **3** | **3** |
| **SEPSIS;TELEMETRY** | **3** | **3** | **6** |
| **SHORTNESS OF BREATH** | **112** | **134** | **246** |
| **STATUS POST MOTOR VEHICLE ACCIDENT WITH INJURIES** | **1** | **5** | **6** |
| **STEMI** | **2** | **4** | **6** |
| **STROKE/TIA** | **6** | **7** | **13** |
| **SUBDURAL HEMATOMA/S/P FALL** | **1** | **5** | **6** |
| **SYNCOPE;TELEMETRY** | **2** | **4** | **6** |
| **SYNCOPE;TELEMETRY;INTRACRANIAL HEMORRHAGE** | **2** | **4** | **6** |
| **TACHYPNEA;TELEMETRY** | **14** | **32** | **46** |
| **TRACHEAL ESOPHAGEAL FISTULA** | **5** | **1** | **6** |
| **TRACHEAL STENOSIS** | **3** | **4** | **7** |
| **UNSTABLE ANGINA** | **4** | **2** | **6** |
| **UPPER GI BLEED** | **6** | **8** | **14** |
| **URINARY TRACT INFECTION;PYELONEPHRITIS** | **3** | **4** | **7** |
| **UROSEPSIS** | **5** | **1** | **6** |
| **UTI/PYELONEPHRITIS** | **2** | **5** | **7** |
| **VARICEAL BLEED** | **1** | **5** | **6** |
| **VF ARREST** | **4** | **2** | **6** |
| **VOLVULUS** | **3** | **4** | **7** |
| **Total** | **1438** | **1870** | **3308** |

**Note:** This table summarizes the frequency of major discharge diagnoses in the MIMIC-III dataset (n=3,308), stratified by sex. Diagnoses include infectious, respiratory, cardiovascular, and metabolic conditions, reflecting the heterogeneity of the external validation cohort.
