# Supplementary material for: Accurate prediction of sepsis from pediatric emergency department to PICU using a machine-learning model
Source: Front Pediatr. 2025 Oct 10;13:1610187. doi: 10.3389/fped.2025.1610187 (PMC12550503; doi:10.3389/fped.2025.1610187)
Supplement: Supplementary file 4 [file Supplementaryfile4.docx]

**Additional File 9.** Computational efficiency and predictive accuracy of interpolation strategies.

| Interpolation  method | Average input features per sample | Training  time(s) | Relative  speed | AUROC(mean  ±SD) |
| --- | --- | --- | --- | --- |
| MGP only | 128 | 310 | 1.0× | 0.832±0.012 |
| CTWH+MGP | 41 | 100 | 3.1× | 0.829±0.014 |
| CTWH only | 38 | 88 | 3.5× | 0.802±0.018 |

**Note:** This table compares multivariate Gaussian process (MGP), correlation-enhanced continuous time-window histogram with MGP (CTWH+MGP), and CTWH alone. CTWH+MGP achieved similar AUROC to MGP alone while significantly reducing input dimensionality and training time, highlighting its practical advantage for real-time clinical application.
