# Supplementary material for: Accurate prediction of sepsis from pediatric emergency department to PICU using a machine-learning model
Source: Front Pediatr. 2025 Oct 10;13:1610187. doi: 10.3389/fped.2025.1610187 (PMC12550503; doi:10.3389/fped.2025.1610187)
Supplement: Supplementary file 5 [file Supplementaryfile5.docx]

**Additional File 10**. Full performance metrics for the XGBoost model across 0–12 h prediction windows.

| Prediction Window (ti, h) | AUROC | Accuracy | Sensitivity | Specificity | Youden Index |
| --- | --- | --- | --- | --- | --- |
| 0 h | 0.842 | 0.803 | 0.784 | 0.818 | 0.602 |
| 2 h | 0.815 | 0.789 | 0.762 | 0.806 | 0.568 |
| 4 h | 0.769 | 0.755 | 0.734 | 0.772 | 0.506 |
| 6 h | 0.728 | 0.721 | 0.702 | 0.740 | 0.442 |
| 8 h | 0.684 | 0.693 | 0.671 | 0.707 | 0.378 |
| 10 h | 0.642 | 0.664 | 0.648 | 0.680 | 0.328 |
| 12 h | 0.611 | 0.637 | 0.623 | 0.650 | 0.273 |

**Note:** This table summarizes AUROC, accuracy, sensitivity, specificity, and Youden index for the XGBoost model in the internal validation cohort. Results demonstrate strong short-term performance but progressively decreasing discriminative ability at longer horizons, consistent with the temporal dynamics observed in the main text (Section 3.3).
