# Supplementary material for: Accurate prediction of sepsis from pediatric emergency department to PICU using a machine-learning model
Source: Front Pediatr. 2025 Oct 10;13:1610187. doi: 10.3389/fped.2025.1610187 (PMC12550503; doi:10.3389/fped.2025.1610187)
Supplement: Supplementary file 6 [file Supplementaryfile6.docx]

**Additional File 12.** Comparative performance of RNN, LSTM, and GRU architectures.

| **Time Before Onset (h)** | **RNN AUROC** | **LSTM AUROC** | **GRU AUROC** |
| --- | --- | --- | --- |
| 0 | 0.890 | 0.892 | 0.891 |
| 2 | 0.870 | 0.873 | 0.872 |
| 4 | 0.840 | 0.844 | 0.843 |
| 6 | 0.810 | 0.815 | 0.814 |
| 8 | 0.780 | 0.784 | 0.784 |
| 10 | 0.740 | 0.743 | 0.743 |
| 12 | 0.720 | 0.722 | 0.722 |

**Note:** AUROC values are shown across prediction horizons (0–12 h before sepsis onset) in the internal validation cohort. LSTM and GRU achieved marginally higher AUROC than RNN, but differences were <0.01, supporting the selection of RNN for efficiency.
