# Supplementary material for: Accurate prediction of sepsis from pediatric emergency department to PICU using a machine-learning model
Source: Front Pediatr. 2025 Oct 10;13:1610187. doi: 10.3389/fped.2025.1610187 (PMC12550503; doi:10.3389/fped.2025.1610187)
Supplement: Supplementary file 7 [file Supplementaryfile7.docx]

**Additional File 16.** Feature Importance in XGBoost Model by Time Window

| Variable | Weight (0–2 h before onset) | Weight (2–12 h before onset) |
| --- | --- | --- |
| Glucocorticoids | 0.207 | 0.196 |
| Lactate | 0.152 | 0.171 |
| Creatinine | 0.141 | 0.162 |
| Heart Rate | 0.119 | 0.112 |
| O2 Saturation | 0.101 | 0.103 |
| WBC Count | 0.093 | 0.091 |
| Respiratory Rate | 0.086 | 0.086 |
| Temperature | 0.056 | 0.048 |
| pH | 0.045 | 0.031 |
| Glucocorticoids | 0.207 | 0.196 |

**Note:** This table shows the SHAP-derived feature weights for the XGBoost model across two time horizons relative to sepsis onset (0–2 h and 2–12 h). Short-term predictions were dominated by therapeutic interventions (glucocorticoids) and acute biomarkers (lactate, creatinine), while longer-term predictions highlighted metabolic stability (lactate, creatinine) and vital signs (HR, SpO₂, RR).
