# Supplementary figures and images for: Accurate prediction of sepsis from pediatric emergency department to PICU using a machine-learning model
Source: Front Pediatr. 2025 Oct 10;13:1610187. doi: 10.3389/fped.2025.1610187 (PMC12550503; doi:10.3389/fped.2025.1610187)

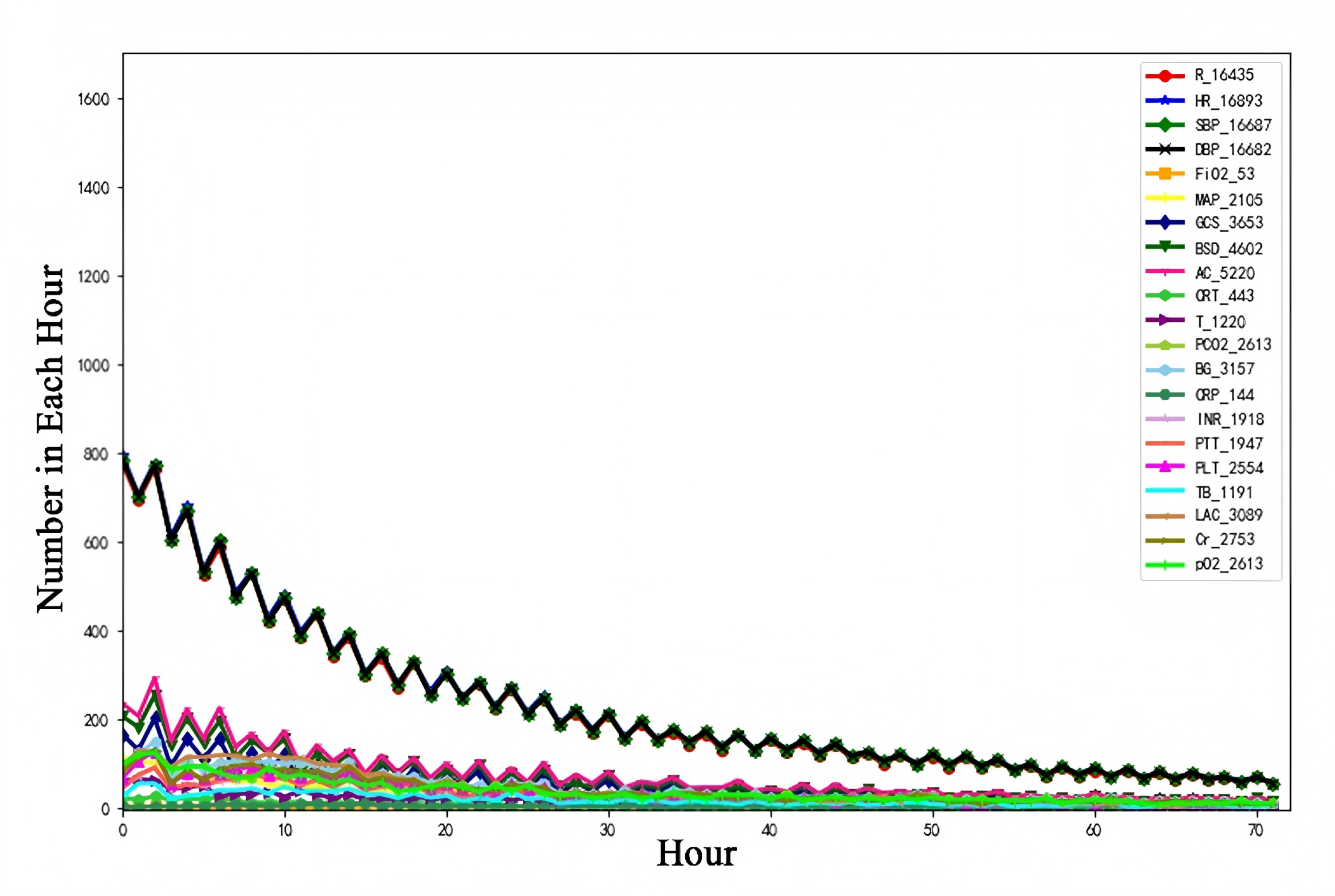

Supplement: Supplementary Figure 1 — Temporal sampling frequency of clinical variables. [file Image1.tiff]

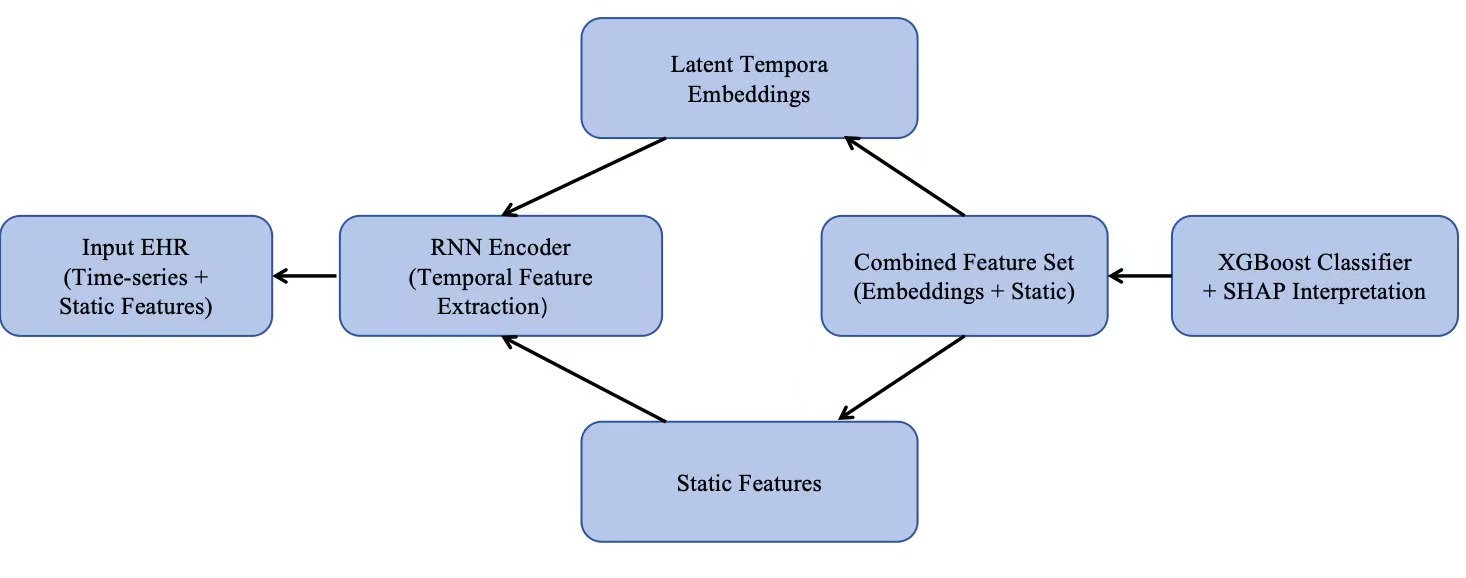

Supplement: Supplementary Figure 2 — Hybrid RNN–XGBoost architecture with SHAP interpretation. [file Image2.tiff]

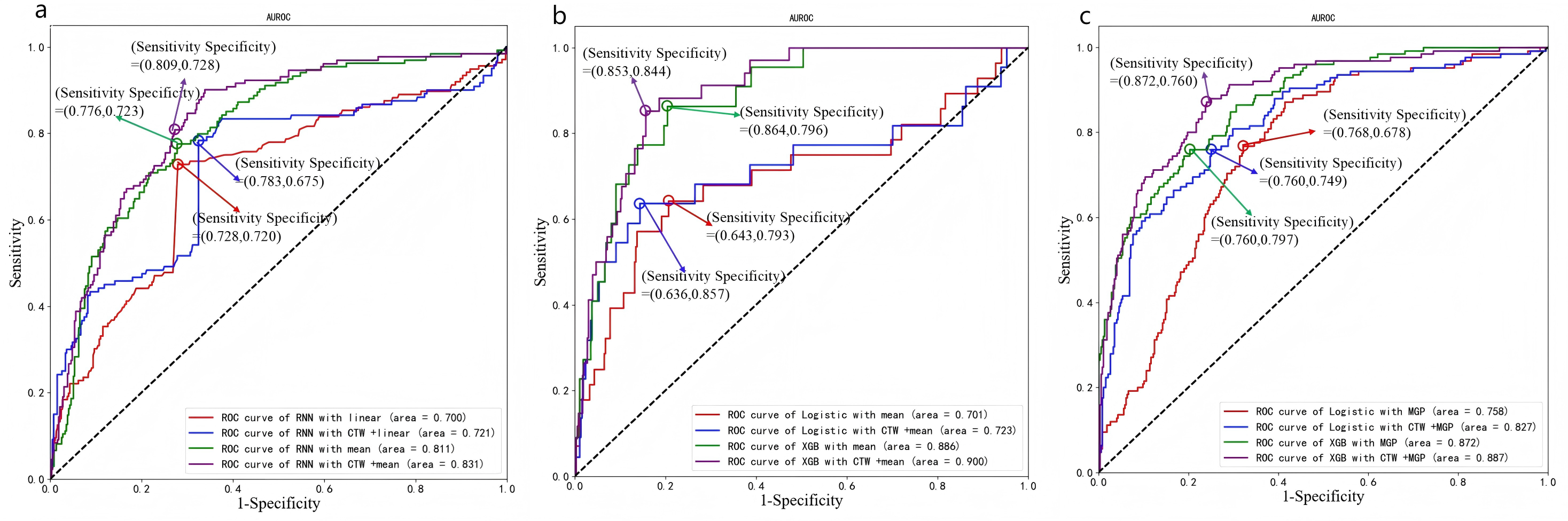

Supplement: Supplementary Figure 3 — Statistical significance heatmap of all candidate features between sepsis and non-sepsis groups. [file Image3.tiff]

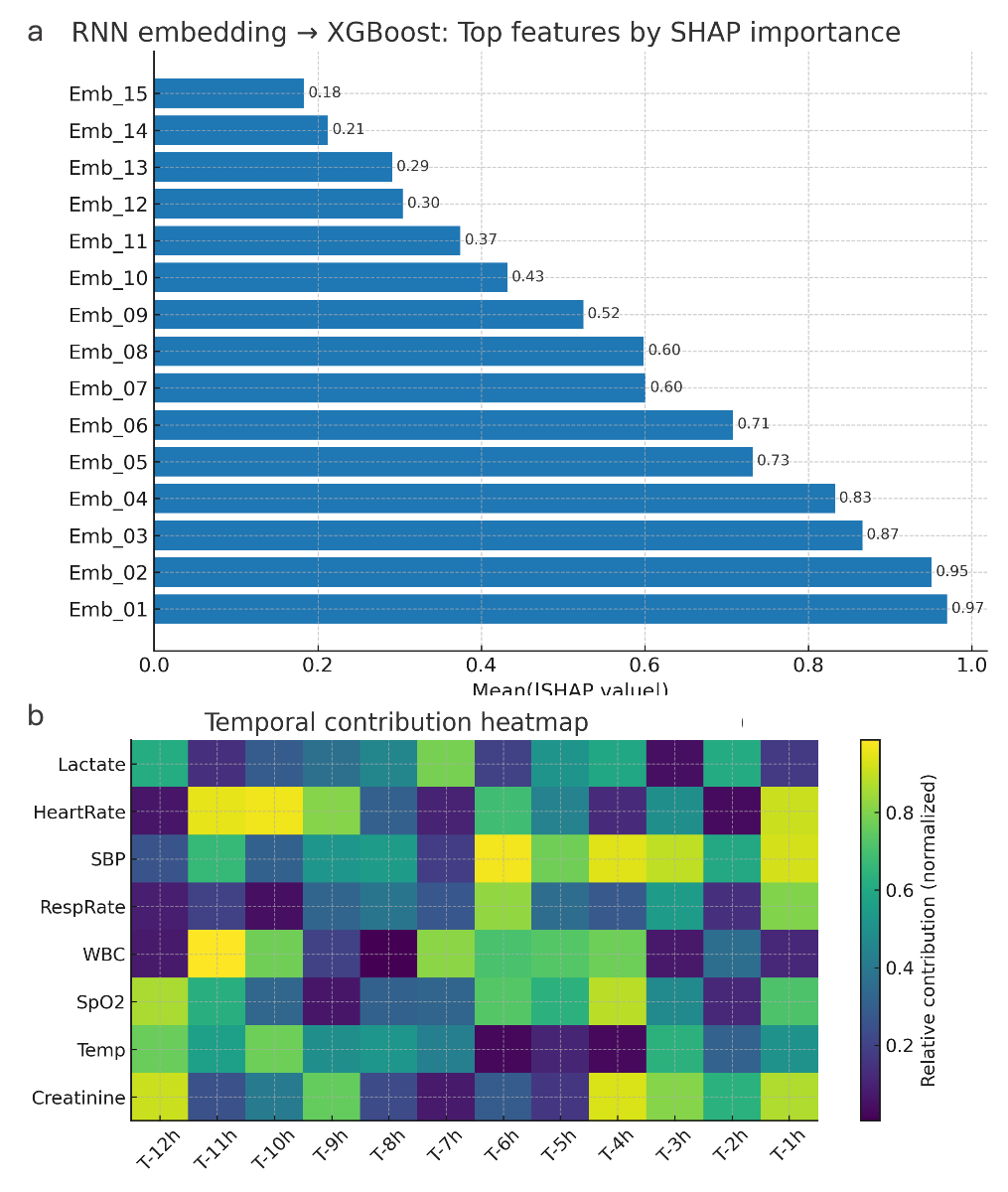

Supplement: Supplementary Figure 4 — Feature importance and temporal contribution analysis using SHAP. [file Image4.tif]

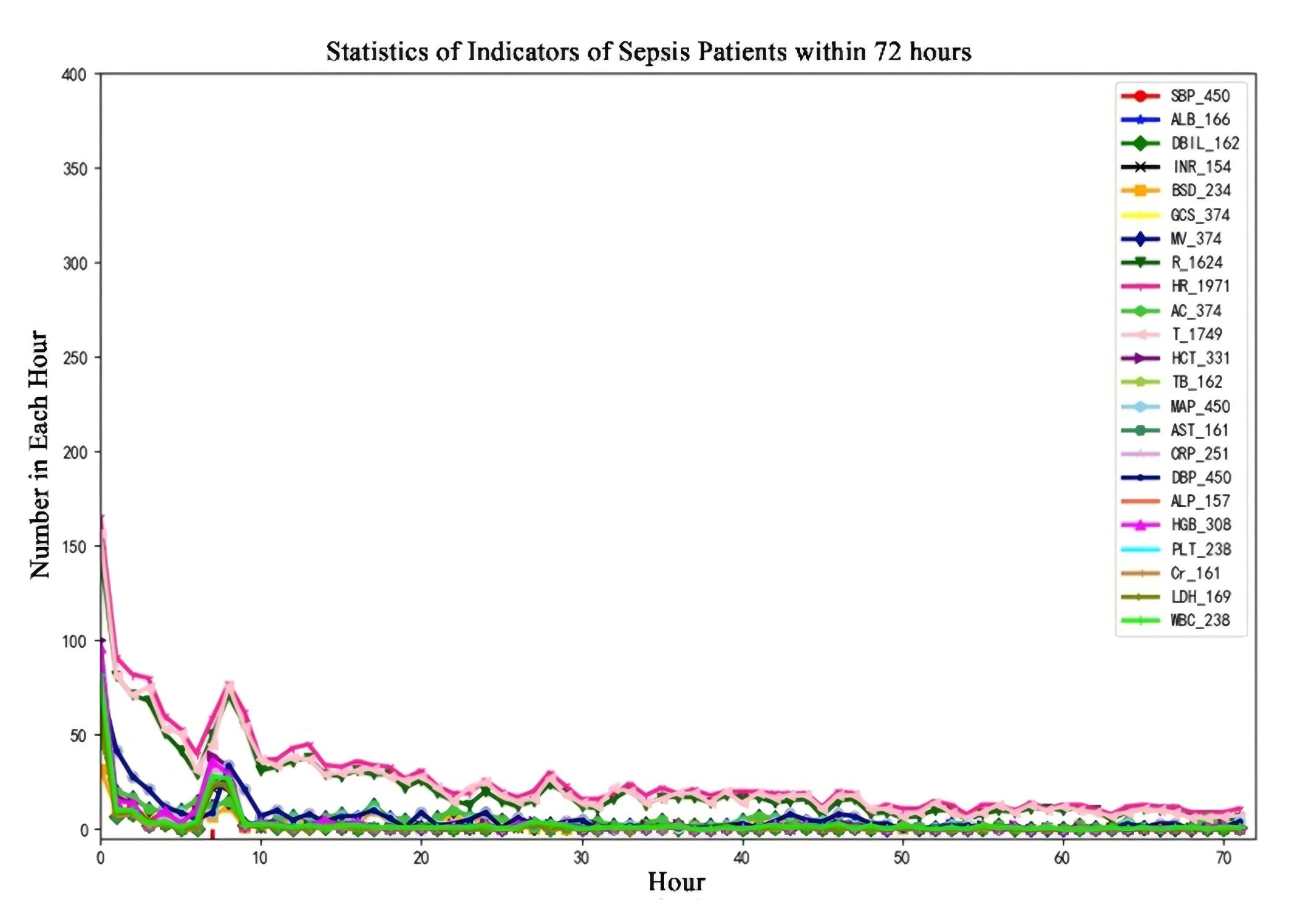

Supplement: Supplementary Figure 5 — Distribution of clinical indicators in sepsis patients within 72 hours of onset. [file Image5.tiff]

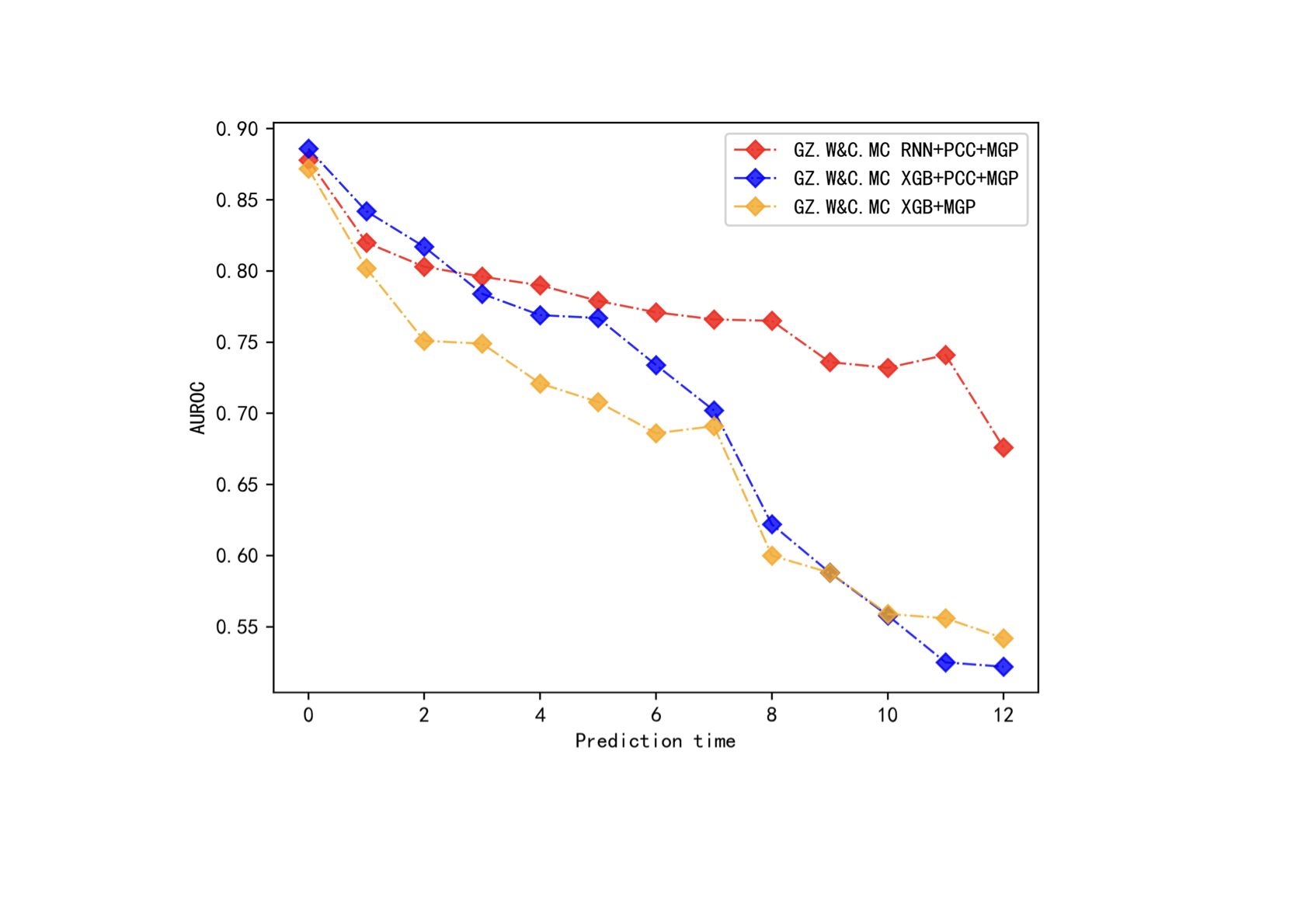

Supplement: Supplementary Figure 6 — Temporal AUROC performance of different model–interpolation combinations. [file Image6.tiff]

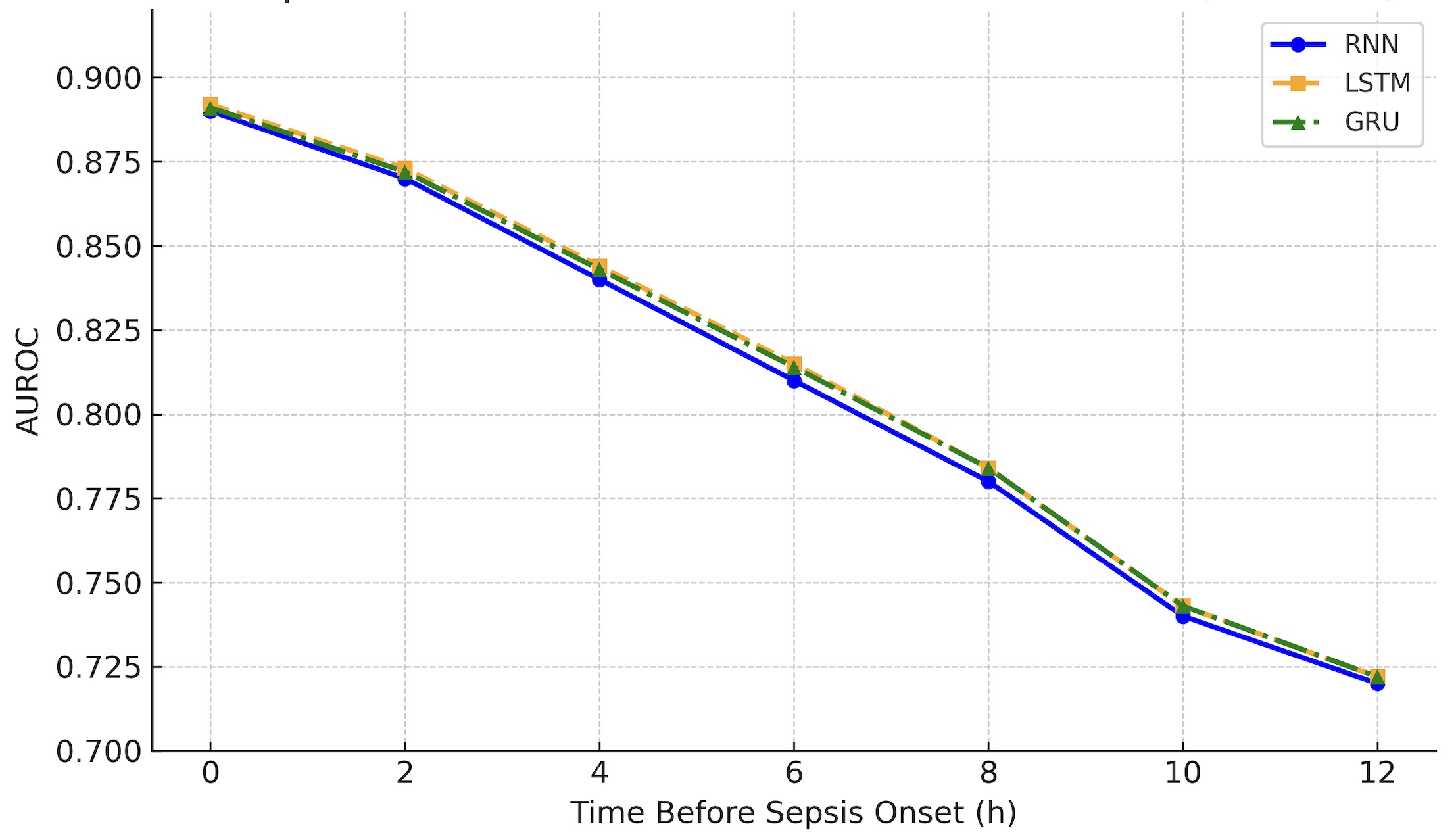

Supplement: Supplementary Figure 7 — AUROC comparison of RNN, LSTM, and GRU models across prediction horizons. [file Image7.tiff]

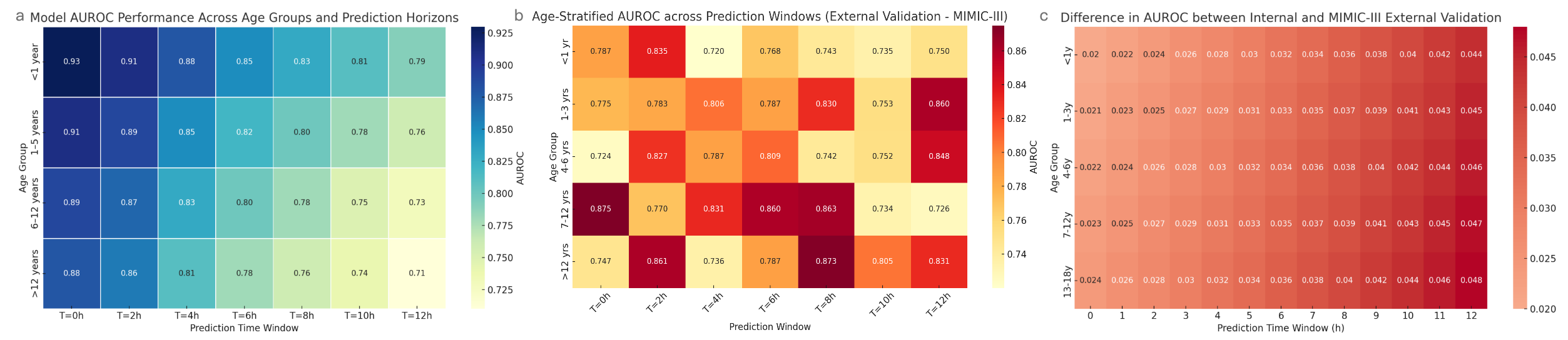

Supplement: Supplementary Figure 8 — Age-stratified AUROC performance across prediction windows. [file Image8.tif]

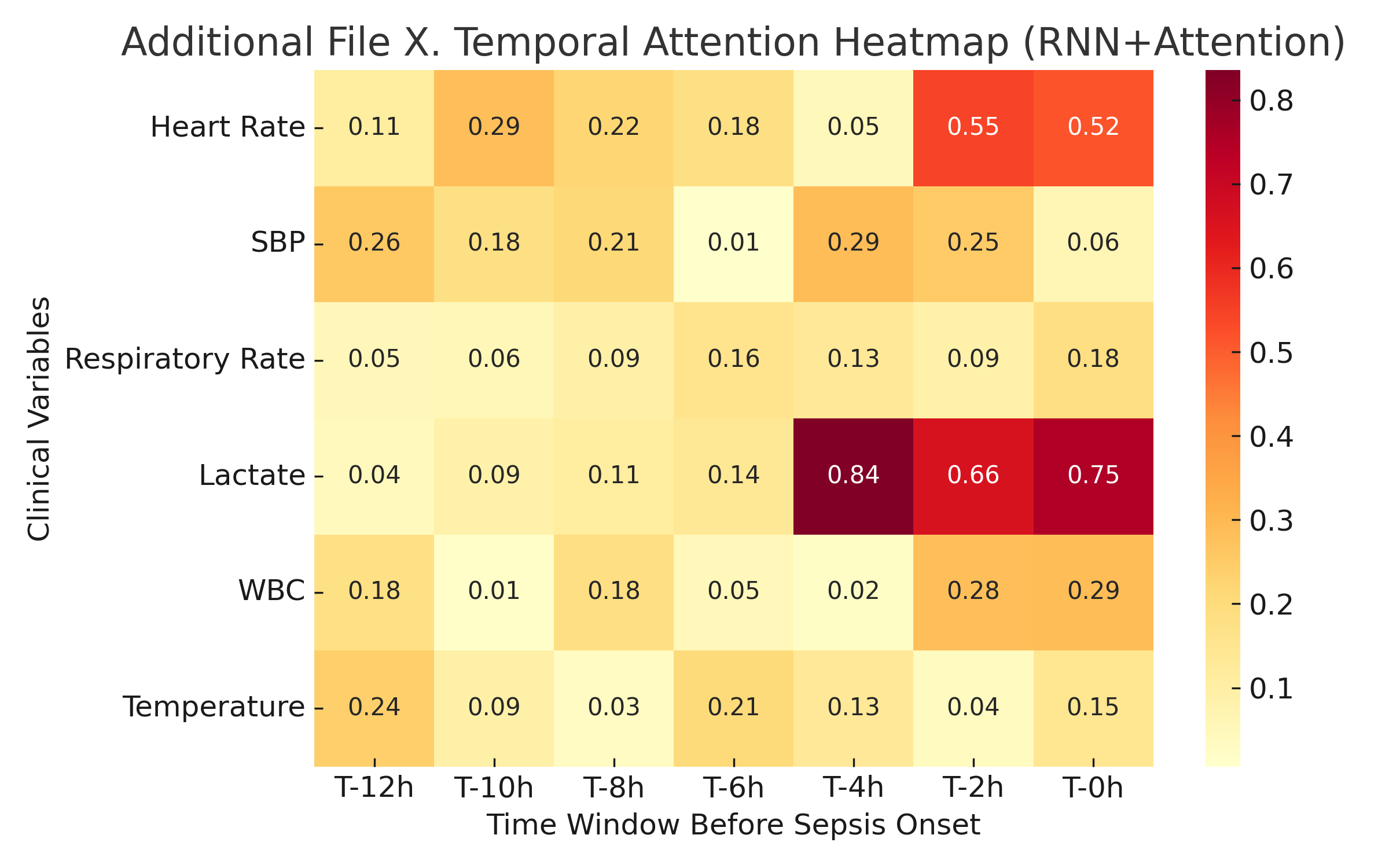

Supplement: Supplementary Figure 9 — Temporal dynamics of variable importance across sepsis prediction windows. [file Image9.tiff]

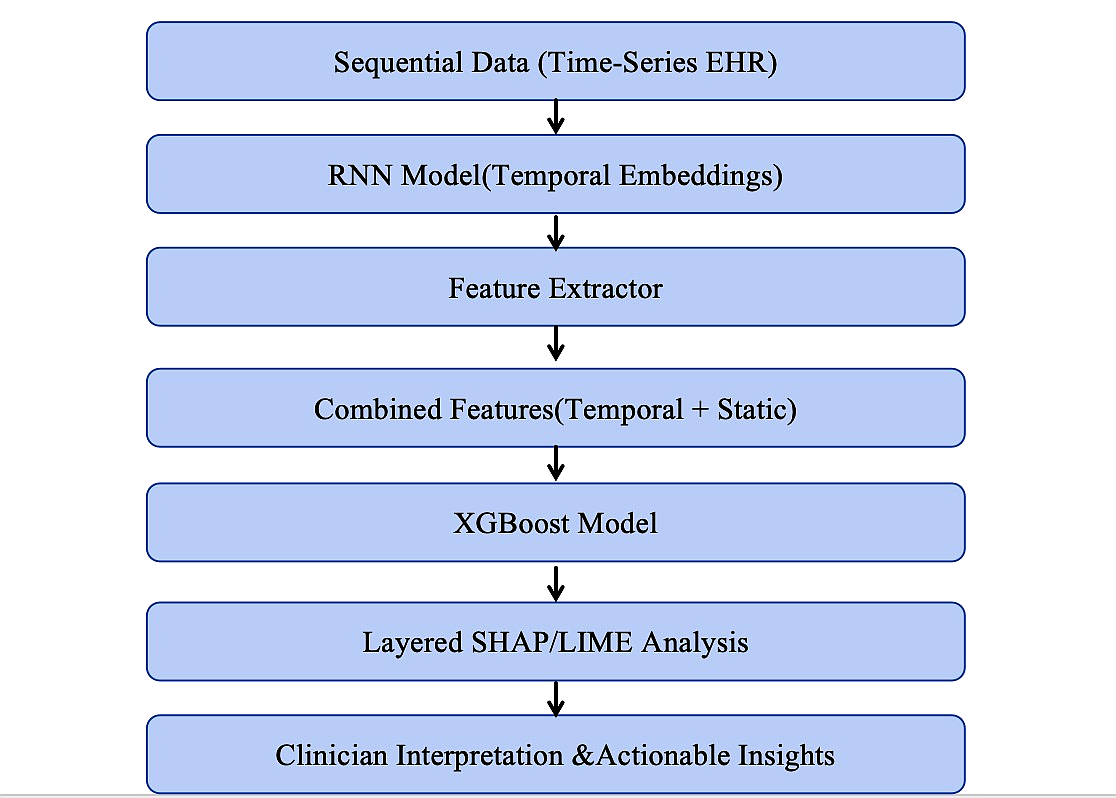

Supplement: Supplementary Figure 10 — Workflow of the layered interpretability pipeline. [file Image10.tiff]

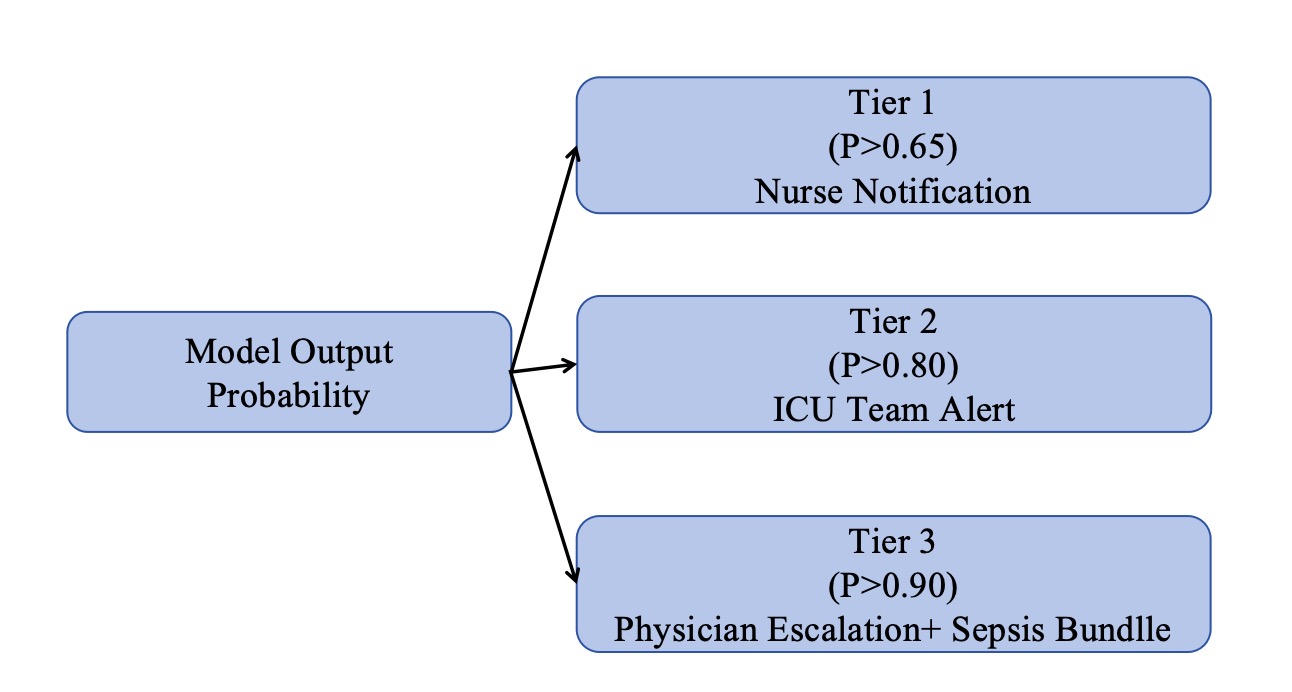

Supplement: Supplementary Figure 11 — Tiered alert system for clinical deployment of the pediatric sepsis prediction model. [file Image11.tiff]
